# Heterogeneity in Regional Damage Detected by Neuroimaging and Neuropathological Studies in Older Adults With COVID-19: A Cognitive-Neuroscience Systematic Review to Inform the Long-Term Impact of the Virus on Neurocognitive Trajectories

**DOI:** 10.3389/fnagi.2021.646908

**Published:** 2021-06-03

**Authors:** Riccardo Manca, Matteo De Marco, Paul G. Ince, Annalena Venneri

**Affiliations:** ^1^Department of Neuroscience, University of Sheffield, Sheffield, United Kingdom; ^2^Department of Life Sciences, Brunel University London, Uxbridge, United Kingdom

**Keywords:** neuroimaging, neuropathology, COVID-19, ageing, stroke, encephalopathy, encephalitis

## Abstract

**Background:** Other than its direct impact on cardiopulmonary health, Coronavirus Disease 2019 (COVID-19) infection affects additional body systems, especially in older adults. Several studies have reported acute neurological symptoms that present at onset or develop during hospitalisation, with associated neural injuries. Whilst the acute neurological phase is widely documented, the long-term consequences of COVID-19 infection on neurocognitive functioning remain unknown. Although an evidence-based framework describing the disease chronic phase is premature, it is important to lay the foundations for future data-driven models. This systematic review aimed at summarising the literature on neuroimaging and neuropathological findings in older over-60 patients with COVID-19 following a cognitive neuroscientific perspective, to clarify the most vulnerable brain areas and speculate on the possible cognitive consequences.

**Methods:** PubMed and Web of Science databases were searched to identify relevant manuscripts published between 1st March 2020 and 31th December 2020. Outputs were screened and selected by two assessors. Relevant studies not detected by literature search were added manually.

**Results:** Ninety studies, mainly single cases and case series, were included. Several neuroimaging and neuropathological findings in older patients with COVID-19 emerged from these studies, with cerebrovascular damage having a prominent role. Abnormalities (hyperintensities, hypoperfusion, inflammation, and cellular damage) were reported in most brain areas. The most consistent cross-aetiology findings were in white matter, brainstem and fronto-temporal areas. Viral DNA was detected mainly in olfactory, orbitofrontal and brainstem areas.

**Conclusion:** Studies on COVID-19 related neural damage are rich and diverse, but limited to description of hospitalised patients with fatal outcome (i.e., in neuropathological studies) or severe symptoms (i.e., in neuroimaging studies). The damage seen in this population indicates acute and largely irreversible dysfunction to neural regions involved in major functional networks that support normal cognitive and behavioural functioning. It is still unknown whether the long-term impact of the virus will be limited to chronic evolution of acute events, whether sub-clinical pathological processes will be exacerbated or whether novel mechanisms will emerge. Based on current literature, future theoretical frameworks describing the long-term impact of COVID-19 infection on mental abilities will have to factor in major trends of aetiological and topographic heterogeneity.

## Introduction

At the end of 2020, the global pandemic of Coronavirus Disease 2019 (COVID-19) has already affected more than 77 million people and caused over 1.8 million deaths worldwide. Although COVID-19 manifests primarily with respiratory problems, the detrimental consequences of this infection may be much wider. A fast-growing body of recent publications has been showing that infection due to COVID-19 may attack multiple organ systems to a variable extent, especially in vulnerable people with prior medical conditions. In particular, older adults are among those most severely affected by the current pandemic and mortality rates have been reported to be particularly high in older populations (Shahid et al., 2020). Possible causes of such increase in vulnerability to the COVID-19 infections include ageing-related changes occurring naturally in the immune system, associated with a reduction in the effectiveness of the immune response (Oh et al., 2019). As a consequence, older adults appear to be more vulnerable than younger adults and children to the cytokine storm activated as a response to the infection (Nidadavolu and Walston, 2020). This older population is also the cohort at greatest risk of neurodegenerative diseases.

The first pathological examinations carried out on patients deceased because of complications associated with COVID-19 showed that signs of this infection extend beyond body tissues directly associated with the respiratory system (Xu et al., 2020). These findings have raised several concerns about the consequences COVID-19 may have on extra-respiratory body systems in older patients, in particular the nervous system. In fact, a variety of neurological complications has been reported in about 25% of patients in some reports (e.g. Romagnolo et al., 2020), even though high variability in symptom prevalence and incidence has been observed across studies (Herman et al., 2020). At present, no evidence-based link exists between COVID-19 and risk of neurodegeneration; however, at this stage it is particularly important to outline a data-driven framework that could inform the study of the long-term neurological consequences of this infectious disease. Since COVID-19 was identified only in December 2019 and declared a pandemic in March 2020, thorough and incessant efforts have been made to prioritise the characterisation of its acute effects on the nervous system. Although studies of the acute effects of COVID-19 are, undoubtedly, a priority, it remains unknown whether the infection and its acute neurological effects play a role as part of long-term neurological trajectories. Acquired neural damage may increase the risk of initiating or worsening neurodegenerative processes (Heneka et al., 2020), possibly in a differential manner depending on the type of neurodegenerative condition (Ferini-Strambi and Salsone, 2020). The study of the effects of COVID-19 on cognitive decline is an area of interest that might become central in the study of the pathophysiological mechanisms of neurodegeneration and in the future management of neurological patients.

Multiple sources of evidence have already been accumulating on the impact of the current pandemic on mental health of older adults both with and without cognitive decline (Manca et al., 2020). A systematic examination of the literature reporting findings on neural damage observed as a consequence of COVID-19 infection in older adults will provide an understanding of its impact on cognitive (and neuropsychiatric) symptoms in this population. In particular, this systematic review focusses on neuroimaging and neuropathology findings from the viewpoint of cognitive neuroscience, in order to inform a theoretical framework that could be used to predict the long-term consequences on cognitive functioning triggered by the virus and its acute neurological manifestation. To do so, we were particularly interested in articles describing the consequences of COVID-related acute neurological events on the brain, and that included details on the regions affected. This was done to elucidate whether some brain regions may show variable degrees of vulnerability to the infection in older adults. Such consideration may provide new insights that could inform prognosis and treatment of the possible consequences of COVID-19 on brain health of older patients.

## Methods

A systematic literature search was carried out in PubMed and Web of Science to identify studies that included neuroimaging and neuropathological examinations of older adults who tested positive for COVID-19. The keywords used to carry out this search were: (1) “COVID-19,” “COVID19,” and “SARS-CoV-2” for the COVID-19 infection; (2) “dementia,” “mild cognitive impairment,” “MCI,” “neurodegeneration,” “neurodegenerative,” “Alzheimer's disease,” “AD,” “FTD,” “frontotemporal dementia,” “older adults,” “ageing,” and “aging” for the populations of interest; (3) “neuropathology,” “autopsy,” “post-mortem,” “neuropathological,” “neuroimaging,” “brain,” “MRI,” “magnetic resonance imaging,” “PET,” “positron emission tomography,” “SPECT,” “Single-photon emission computed tomography,” “neuroradiology,” “neuroradiological,” “nuclear medicine,” “stroke,” “ischaemia,” “ischaemic,” “ischemia,” “ischemic,” “vascular,” “encephalitis,” “meningitis,” “vasculitis,” and “encephalopathy” for the neuroimaging/neuropathological variables of interest. Papers published between March 2020 and 31st December 2020 (last day of literature search) were included. All publication entries resulting from the initial search were screened to identify papers reporting original data, with no restrictions on the type of article.

Inclusion criteria were defined as follows: original data describing changes to the nervous tissue associated with COVID-19 infection. The intent was to focus on studies mentioning or illustrating the regional properties of neural abnormalities in order to inform the theoretical basis of a model of cognitive dysfunction due to brain damage or increased vulnerability associated with the virus. Two partially distinct sets of exclusion criteria were then defined to identify eligible studies based on neuroimaging and neuropathology, respectively. Due to the “*intra-vitam*” and routine nature of neuroimaging procedures, exclusion criteria for neuroimaging evidence were defined according to the following four principles: (1) manuscripts not in English or not having completed a peer-review process; (2) manuscripts based on study participants whose inclusion was not associated with COVID-19 infection; (3) studies not distinctively focussing on adults older than 59; (4) studies not including adequate information on how regional properties of brain tissue were affected. Exclusion criterion 2 served to discard all studies run “in the era/at the time of COVID-19” not directly focussing on the physiological effects of the virus, or studies exploring the indirect effects of COVID-19-related factors, e.g., those triggered by lockdown or social-limitation policies. Exclusion criterion 3 was introduced to limit the remit of the review to adults typically defined as “older adults” by neurological studies. By doing so, single-cases of adults aged 59 or less were excluded and case-series were filtered to retain only patients meeting inclusion criteria. Similarly, cohort studies were discarded when no clear age group meeting criteria was identifiable or when the central-tendency and dispersion measures for the “age” variable were suggestive of a sample excessively skewed towards a younger age or excessively heterogeneous. Finally, exclusion criterion 4 was set to discard manuscripts not exploring or investigating the brain, as well as manuscripts describing cerebrovascular abnormalities (e.g., as informed by angiographic scans) without a specific focus on damage of the nervous tissue. Exclusion criteria for neuropathology studies were the same as above with the exception of criterion 3. Given the unique nature of neuropathological studies, no age-based exclusion was applied.

Two independent assessors (MDM and RM) reviewed the search output to process each entry and either exclude or retain it. A third assessor (AV) helped resolving any disagreement on publications to be included. Additional papers relevant to this review identified through other sources (i.e., references and key journals) were also screened and manually added.

## Results

**Figure 1:**
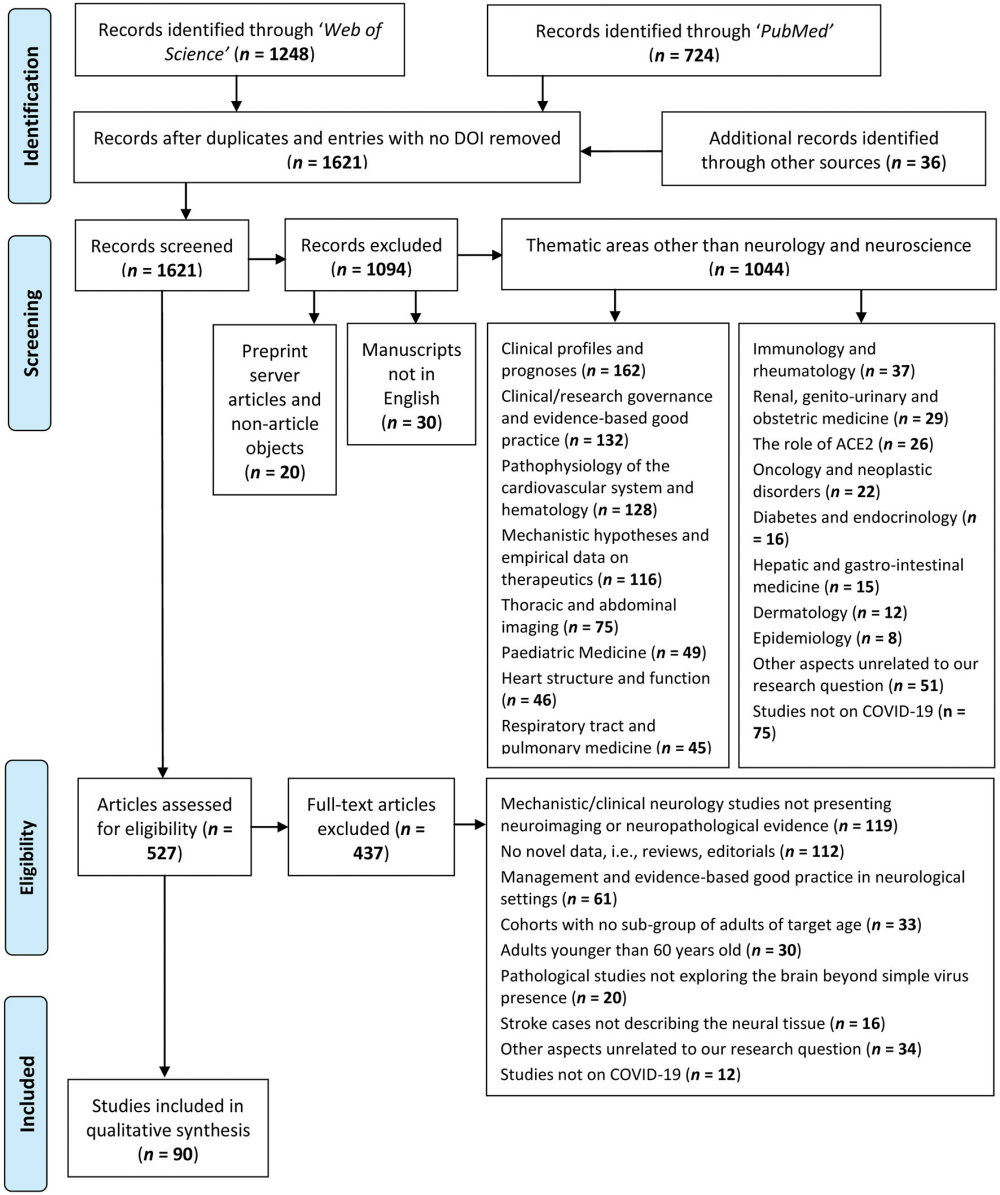
Flow chart outlining the study selection process according to the PRISMA framework.

A flow diagram illustrating the process of manuscript inclusion is reported in Figure 1. The above search strings resulted in a total of 1,972 articles. After removal of duplicates and objects with no digital object identifier (DOI), 1,621 elements were retained, 50 of which were immediately discarded. These included manuscripts not in English (*n* = 30), manuscripts deposited in pre-print servers and not having yet completed a process of peer review (*n* = 3) and non-article objects (i.e., figures, tables, and data sheets) that had their own DOI (*n* = 17). The remaining manuscripts were screened and separated according to the central medical specialty of reference (reported in Figure 1). Following this classification, 527 manuscripts on neurological or neurology-related themes (e.g., cardiological studies including reference to the cerebrovascular system or articles of mixed neurological-psychiatric interest) were retained and assessed for study eligibility. In addition, all pathology-related studies were also included in the list shortlisted for study eligibility since in this first year of COVID-19, pathological studies have investigated a wide-range of *post-mortem* tissues (including the brain) in a more general rather than specialised way. Following the procedures of assessment, 437 of the 527 manuscripts were excluded. These were categorised based on the reason behind failed suitability (see Figure 1 for a complete list). In particular, 16 studies were excluded because, although describing patients with stroke, they limited their description to the cerebrovascular accidents without focussing on the damage to the neural tissue. Based on the same principle, 20 studies of pathology were discarded because they did not describe properties of the neural tissue, but instead limited the investigation to other organs or to aspects relevant to the nervous system other than tissue involvement (e.g., analysis of cerebrospinal fluid). Similarly, pathological studies that solely investigated the presence of the virus were not considered. As a result, 90 articles met study eligibility criteria and were thus included in this systematic review. These mainly included single-case reports and case series plus a small number of group studies (a summary for each article is reported in Table 1).

**Table 1:** Summary of the characteristics and findings of the studies included in the review.

**Study**	**Design**	***N***	**Age**	**Sex (F/M)**	**Aetiology**	**Comorbidities**	**Method**	**Country**	**Brain findings**
**Neuroimaging findings**									
Anand et al. (2020)	Case series	5	61, 75, 81, 88, 88	4/1	Encephalopathy - seizures	TBI, remote left MCA infarct, PD, history of cardiac arrest, end-stage renal disease, intellectual disability	CT	USA	CT abnormalities in left frontal, parietal, and temporal lobes (and in left MCA territory, due to a prior infarct); right frontal and bilateral cerebellar leukoencephalopathy and gyral diffusion alterations; no abnormalities in two cases.
Delorme et al. (2020)	Case series	4	60, 66, 69, 72	2/2	Encephalopathy	None reported	MRI and FDG-PET	France	Hypometabolism in bilateral frontal cortices in all cases (prefrontal in three and orbitofrontal in one case) and in posterior associative cortices in two cases (only left parieto-temporal in one case); hypermetabolism in the cerebellar vermis in all, bilateral striatum in two cases. In one case, right orbitofrontal hyperintensities,
Fernández-Domínguez et al. (2020)	Single case	1	74	1/0	Encephalopathy—Miller-Fisher-like syndrome	Hypertension and follicular lymphoma treated from 2014 to 2015	MRI	Spain	No abnormalities.
Guedj et al. (2020)	Single case (#2 from a case series)	1	62	0/1	Encephalopathy	No significant prior conditions	FDG-PET (whole body)	France	Hypometabolism in: bilateral medial temporal lobe, cerebellum, hypothalamus, left thalamus, right gyrus rectus, medulla oblongata, pons, left cingulate gyrus and right precentral, postcentral and superior temporal gyri.
Jang et al. (2020)	Single case	1	67	0/1	Encephalopathy	Anorexia and depression	CT and MRI	USA	No abnormalities on CT; mild scattered deep periventricular and subcortical WM ischaemic lesions on MRI, but no evidence of encephalitis, posterior reversible encephalopathy, or leukoencephalopathy.
Logmin et al. (2020)	Single case	1	70	1/0	Encephalopathy - recurrent non-epileptic seizures/convulsive syncope	Syncope, neuropathic pain, paroxysmal atrial fibrillation	MRI	Germany	No abnormalities, apart from three hyperintensities due to minimal prior ischaemic events.
Manganelli et al. (2020)	Case series	2	66, 67	1/1	Encephalopathy	None reported	CT and MRI	Italy	No MRI abnormalities in male patient; scattered gliosis in right pons on CT in one case.
Palomar-Ciria et al. (2020)	Single case	1	65	0/1	Encephalopathy	Schizophrenia	CT and MRI	Spain	Deep WM leukoencephalopathy due to small vessel pathology on CT (unclear relation to COVID-19); dilatation of ventricles and subarachnoid spaces in line with the patient's age on MRI.
Vollono et al. (2020)	Single case	1	78	1/0	Encephalopathy—non-convulsive status epilepticus	Hypertension, epilepsy due to prior Herpes Simplex Virus-1 encephalitis	CT and MRI	Italy	No abnormalities on CT; old gliosis and atrophy involving the left temporal/parietal lobes on MRI, but no recent acute lesions.
Young et al. (2020)	Single case	1	≥ 60	0/1	Encephalopathy—Creutzfeldt-Jakob disease	None reported	MRI and FDG-PET	USA	Hyperintensities and hypometabolism diffuse throughout the left hemisphere cortex, the left caudate nucleus and thalamus and the right cerebellum.
Muccioli et al. (2020)	Case series	4 (out of 5)	75, 69, 69, 67	1/3	Encephalopathy	Type 2 diabetes, hypertension, ischaemic heart disease, previous stroke, MCI, bipolar disorder, iatrogenic parkinsonism, hypertensive cardiopathy	MRI	Italy	Encephalopathy developed after sedation in two patients who showed chronic cerebral small vessel disease; cerebral atrophy and non-specific diffuse parietal WM hyperintensity in one case; old right fronto-parietal stroke in one case.
Parauda et al. (2020)	Case series	4	64, 73, 65, 74	2/2	Encephalopathy	Hypertension, diabetes, hypothyroidism, hyperlipidaemia	CT and MRI	USA	#1: CT at admission was unremarkable, but new bilateral occipital confluent WM hypodensities and lucencies in fronto-parietal WM and in left posterior limb of the internal capsule after 6 days; MRI-confirmed hyperintensities in same locations after 32 days. #2, #3, #4: hypoattenuation in bilateral parietal-occipital WM on CT and hyperintensities in same areas on MRI.
Pugin et al. (2020)	Case series	5	75 (69-78)^a^	2/3	Encephalopathy	Hypertension, diabetes, smoking, immunodepression, COPD, chronic kidney disease, cerebrovascular disease	MRI	Switzerland	All patients under mechanical ventilation. Abnormal contrast enhancement, consistent with inflammation of endothelial cells, in vascular walls of: vertebral artery (all cases), internal carotid (three cases), basilar artery (two cases) and both PCAs (one case); bilateral small watershed ischaemia in one case; no other brain abnormalities or enhancements in leptomeningeal spaces.
Chaumont et al. (2020)	Single case	1	69	0/1	Encephalitis—meningoencephalitis	None reported	MRI	France - Guadeloupe	No abnormalities.
Hosseini et al. (2020)	Single case (#2 from a case series)	1	79	1/0	Encephalitis—limbic encephalitis	None reported	CT and MRI	UK	Chronic small vessel ischaemic damage on first MRI; diffusion alterations in mediotemporal and limbic areas on subsequent CT and MRI.
Khoo et al. (2020)	Single case	1	65	1/0	Encephalitis—brainstem encephalitis	Osteoarthritis and gastro-oesophageal reflex disease, suspected AD	MRI	UK	No abnormalities.
Le Guennec et al. (2020)	Single case	1	69	0/1	Encephalitis—orbitofrontal encephalitis	Diabetes, hypertension, one previous seizure	CT and MRI	France	No abnormalities on CT; hyperintensity in the right orbitofrontal cortex, mesial prefrontal cortex and caudate nucleus. The hyperintensity persisted in the right caudate after 15 days, but completely resolved after 30 days.
Novi et al. (2020)	Single case	1	64	1/0	Encephalitis—ADEM	Vitiligo, hypertension, and monoclonal gammopathy	MRI	Italy	ADEM characterised by gadolinium-enhancing lesions in spinal cord, optic tract and in temporal/ occipital and frontal areas.
McCuddy et al. (2020)	Single case (#3 from a case series)	1	70	1/0	Encephalitis—ADEM	Obesity, peripheral neuropathy, glaucoma, type 2 diabetes, hypertension, chronic kidney disease, hyperlipidaemia	MRI	USA	Hyperintense lesions, mostly with restricted diffusion, in deep WM, corpus callosum and left brachium pontis. Slight improvement after 8 days.
Pilotto et al. (2020)	Single case	1	60	0/1	Encephalitis	None reported	CT and MRI	Italy	No abnormalities.
Avula et al. (2020)	Case series	4	73, 83, 80, 88	3/1	Cerebrovascular—ischaemia	Hypertension, dyslipidaemia, carotid stenosis, frequent urinary tract infections, type 2 diabetes and neuropathy	CT and MRI	USA	#1: Left parieto-occipital territory; #2: Right posterior frontal lobe; #3: Right middle-cerebral-artery stroke with hypoperfusion extending to almost the entire hemisphere; #4: Left mediotemporal lobe.
Basi et al. (2020)	Single case	1	66	0/1	Cerebrovascular—ischaemia	COPD, atrial fibrillation and previous ischaemic stroke	CT	UK	Right inferior medial prefrontal lobe with suspected infarction in the right cerebellum.
Katz et al. (2020)	Single case (from a case series)	1 (with neuroimaging details out of 86 cases)	62	1/0	Cerebrovascular—ischaemia	None reported	CT	USA	Bilateral middle cerebral artery infarction with anterior frontal involvement.
Morassi et al. (2020)	Case series	4 from a series of 6 cases	64, 75, 82, 76	1/3	Cerebrovascular—ischaemia	History of smoking, history of myocardial infarction, hypertension, diabetes mellitus, previous TIA, previous stroke, aortic valve replacement	CT	Italy	#1: Various cortical and subcortical regions of both hemispheres (including left occipital and right precentral territory); #2: Right cingulate gyrus, right fronto-parietal, left pericentral, bilateral occipital and vermian/left cerebellar areas; #3: Right thalamus and right temporal centrum semiovale; #4: Right caudate, left prerolandic and superior frontal areas.
Zayet et al. (2020)	Case series	2	84, 74	0/2	Cerebrovascular—ischaemia	Diabetes mellitus, arterial hypertension, coronary heart disease, peripheral arterial disease and atrial fibrillation, multiple cardiovascular diseases (including atrial fibrillation)	MRI	France	#1: Multiple regions including bilateral cerebellum, right occipital cortex, bilateral parieto-occipital cortical territory and fronto-parietal subcortical regions; #2: Large left frontal ischaemia and additional ischaemic areas in the cerebellum and in the parieto-occipital cortex, bilaterally.
Barrios-López et al. (2020)	Case series	3 from a series of 4 cases (#2, #3, #4)	64, 85, 87	2/1	Cerebrovascular—ischaemia	Hypertension, type 2 diabetes, hypertensive heart disease, asthma, atrial fibrillation and ischaemic heart disease	CT	Spain	#2: Left cerebellar and occipito-temporal regions; #3: Right fronto-temporal regions; #4: Right middle cerebral artery territory.
Mohamud et al. (2020)	Case series	4 from a series of 6 cases (#2, #3, #4, #6)	78, 62, 74, 67	1/3	Cerebrovascular—ischaemia	Diabetes, hypertension, chronic kidney disease and hyperlipidaemia	CT	USA	#2: Left caudate, putamen, and left fronto-parietal and paracentral cortices; #3: Right frontal and temporal lobes; #4 and #6: No abnormalities.
Papi et al. (2020)	Single case	1	79	1/0	Cerebrovascular—ischaemia	Hypertension, ischaemic heart disease, type 2 diabetes and atrial fibrillation	CT	Italy	Left frontal, parietal, insular and temporal areas of penumbra.
Bolaji et al. (2020)	Single case	1	63	0/1	Cerebrovascular—ischaemia	Diabetes and asthma	CT	UK	Right parietal cortex.
Goldberg et al. (2020)	Single case	1	64	0/1	Cerebrovascular—ischaemia	Hypertension, aplastic anaemia and splenectomy	CT	USA	Bilateral fronto-parietal regions.
Tunç et al. (2020)	Case series	3 from a series of 4 cases (#2, #3, #4)	67, 72, 77	1/2	Cerebrovascular—ischaemia	Hypertension	MRI	Turkey	#2: In proximity to the caudate body; #3: Left fronto-parietal regions; #4: Right pons.
Viguier et al. (2020)	Single case	1	73	0/1	Cerebrovascular—ischaemia	None reported	CT and MRI	France	Left fronto-parietal regions.
Zhang et al. (2020)	Case series	3	69, 65, 70	1/2	Cerebrovascular—ischaemia	Hypertension, diabetes and stroke, coronary artery disease, emphysema and nasopharyngeal carcinoma	CT	China	#1: Frontal, parietal and occipital lobe, basal ganglia, brainstem and cerebellum (bilaterally); #2: Right frontal and bilateral parietal lobe; #3: Bilateral frontal, right parietal, temporal and occipital lobe, and bilateral cerebellar hemispheres.
Diaz-Segarra et al. (2020)	Case series	2 from a series of 4 cases (#3 and #4)	65, 68	1/1	Cerebrovascular—ischaemia	Hypertension and type 2 diabetes	MRI	USA	#3: Scattered punctuated foci in both cerebral hemispheres; #4: Right medial occipital lobe.
Janjua and Moscote-Salazar (2020)	Single case	1	65	1/0	Cerebrovascular—ischaemia	Diabetes and mild dementia	CT	Colombia	Bilateral basal ganglia, occipital lobes and cerebellar hemispheres.
Co et al. (2020)	Single case	1	62	1/0	Cerebrovascular—ischaemia	Hypertension, prediabetes, dyslipidaemia and history of TIA	CT	Philippines	Left centrum semiovale and corona radiata.
Zhai et al. (2020)	Single case	1	79	0/1	Cerebrovascular—ischaemia	Atrial fibrillation	CT	China	Lacunar infarctions at the level of the insula, bilaterally, hippocampus and anterior temporal lobe, bilaterally.
Sparr and Bieri (2020)	Case series	2 from a series of 4 cases (#1 and #3)	84, 62	2/0	Cerebrovascular—ischaemia	Hypertension and diabetes mellitus	CT and MRI	USA	#1: Splenium of the corpus callosum; #3: Multiple bilateral cerebral and cerebellar infarctions and the right side of the splenium of the corpus callosum.
Jillella et al. (2020)	Case series	10 from a sample of 13 (#2, #3, #5, #6, #7, #8, #9, #11, #12, #13)	8 in their 60's, 2 in their 70's	1/9	Cerebrovascular—ischaemia	Atrial fibrillation or flutter, hypertension, hyperlipidaemia, diabetes, deep venous thrombosis/pulmonary embolism	CT and MRI	USA	#2: Left parietal, right frontal and occipital lobe, bilaterally; #3: Right insula; #5: Left frontal and temporal lobe, bilaterally; #6: Left parieto-occipital; #7: Left temporo-parietal; #8: Right frontal, temporal and parietal; #9: Right thalamus, left cerebellum and left capsula; #11: Left frontal; #12: Basal ganglia, cerebellum and parieto-occipital lobe, bilaterally; #13: fronto-parietal regions.
Kananeh et al. (2020)	Single case (from a case series)	1 from a sample of 4 (#2)	70	1/0	Cerebrovascular—ischaemia	Atrial fibrillation (new onset)	CT	USA	The majority of the right hemisphere.
Tiwari et al. (2020)	Case series	8 from a sample of 16 (#8, #9, #11, #12, #13, #14, #15, #16)	73, 82, 80, 74, 60, 62, 64, 67	4/4	Cerebrovascular—ischaemia	Hypertension, previous cerebrovascular accident, diabetes mellitus, chronic kidney disease, coronary artery disease, congestive heart failure	CT and MRI	USA	#8: Left parieto-occipital; #9: Left frontal; #11: Basal ganglia and capsula; #12: Thalamus and capsula; #13: Capsula; #14: Left putamen; #15: Unspecified right territory; #16: Left parieto-occipital.
Ghani et al. (2020)	Single case (from a case series)	1 out of 3 cases	61	0/1	Cerebrovascular—haemorrhage	Diabetes	CT	USA	Scattered subarachnoid haemorrhages and a subdural hematoma involving the cerebellum.
Benger et al. (2020)	Single case (from a case series)	1 out of 5 cases	54	1/0	Cerebrovascular—haemorrhage	None reported	CT and MRI	UK	Posterior division of the right capsule.
Keaney and Mumtaz (2020)	Single case (from a case series)	1 out of 2 cases	72	1/0	Cerebrovascular—haemorrhage	Hypertension, type 2 diabetes, mild asthma	CT	UK	Extensive damage to the right hemisphere including frontal, temporal and parietal lobes.
Sharifi-Razavi et al. (2020)	Single case	1	79	0/1	Cerebrovascular—haemorrhage	None reported	CT	Iran	Extensive damage in the right temporal lobe.
Roy-Gash et al. (2020)	Single case	1	63	1/0	Cerebrovascular—haemorrhage	None reported	CT and MRI	France	Bilateral temporal.
Al-Dalahmah et al. (2020)	Single case	1	73	0/1	Cerebrovascular—haemorrhage	Hypertension, type 2 diabetes	CT	USA	Large portion of the cerebellum.
Muhammad et al. (2020)	Single case	1	60	1/0	Cerebrovascular—haemorrhage	None reported	CT	Germany	Ruptured aneurysm with damage of left ventromedial prefrontal cortex.
Fitsiori et al. (2020)	Case series	7 out of 9 cases (#A, #C, #D, #F, #G, #I, #J)	66, 76, 78, 79, 65, 72, 62	1/6	Cerebrovascular—haemorrhage	COPD, human immunodeficiency virus, Waldenstrom macroglobulinemia, coronary artery disease, cardiac valvulopathy, hypertension, hypercholesterolemia, prostate cancer, diabetes, dyslipidaemia, sleep apnoea, MCI, vitiligo and obesity	MRI	Switzerland	#A: Microbleeds in subcortical white matter, corpus callosum, basal ganglia, right anterior limb of the anterior capsule and left middle cerebellar peduncle; #C: Microbleeds in the corpus callosum, subcortical white matter and left parietal lobe; #D: Microbleeds in subcortical white matter, corpus callosum, left middle cerebellar peduncle and lacunar infarct in the external capsule; #F: Microbleeds in subcortical white matter and corpus callosum; #G: Lacunar infarcts in subcortical white matter, microbleeds in corpus callosum, middle cerebellar peduncle, posterior limb of the internal capsule, subcortical white matter and pontine myelinolysis; #I: Infarct in the centrum semiovale, microbleeds in the corpus callosum, subcortical white matter, posterior limb of the internal capsule, left middle cerebellar peduncle and cerebellum; #J: Microbleeds in corpus callosum and posterior limb of the internal capsule.
Pavlov et al. (2020)	Case series	2 from a sample of 3 (#2, #3)	64, 60	0/2	Cerebrovascular—haemorrhage	Hypertension, smoking history, type 2 diabetes, type 1 diabetes, hyperlipidaemia	CT	Russia	#2: Right basal ganglia, capsula; #3: Right ganglia, capsula, posterior temporal.
Sabayan et al. (2021)	Single case (from a case series)	1 out of 15 cases (#9)	60	0/1	Cerebrovascular—haemorrhage	Hypertension	CT	Iran	Parietal lobe, bilaterally.
Radmanesh et al. (2020a)	Retrospective database analysis	242 (n = 6 with neuroimaging description: #1, #2, #3, #4, #5, #6)	68.7 (16.7)^b^ (74, 61, 62, 77, 63, 78)	92/150 (2/4)	Cerebrovascular—haemorrhage (#1, #2), ischaemia (#3, #4, #5, #6)	Not systematically described (#1: stented carotid artery, #2: hepatic cirrhosis)	CT and MRI	USA	#1: Right temporal lobe; #2: Left superior parietal regions; #3: Left inferior frontal regions; #4: Right-sided damage extending to the frontal and temporal lobe, capsula and basal ganglia; #5: Left lateral cerebellum; #6: Cingulate gyrus and body of the corpus callosum.
Hernández-Fernández et al. (2020)	Retrospective database analysis	12 from a sample of 23 (#2, #4, #5, #8, #10, #11, #12, #19, #20, #21, #22, #23)	83, 65, 75, 76, 62, 86, 65, 69, 61, 64, 68, 66	1/11	Cerebrovascular—ischaemia (#2, #4, #5, #8, #10, #11, #12), ischaemia and haemorrhage (#19, #21), haemorrhage (#20, #22), encephalopathy and haemorrhage (#23)	Hypertension, dyslipidaemia, ischaemic cardiopathy, rheumatic valve disease and atrial fibrillation, smoking, schizophrenia, type 2 diabetes, COPD, vitamin B12 deficiency, stable angina, sleep apnoea	CT and MRI	Spain	#2: Bilateral cerebellum, left thalamus and occipital regions; #4: Right fronto-temporal regions; #5: Right parietal regions, thalamus and left frontal lobe; #8: Right insula; #10: Cerebellum; #11: Left insula; #12: Right parietal lobe; #19: Extensive left frontal and small right frontal haemorrhages; Bilateral parieto-occipital FLAIR hyperintensities; #20: Left lateral temporal extending to the Sylvian fissure; #21: Multiple foci of cortical-subcortical and subarachnoid haemorrhage in temporal and occipital regions; Bilateral parieto-occipital and cerebellar hyperintensities; #22: Left ventrolateral prefrontal regions and right parieto-occipital white matter; #23: Leukoencephalopathy in the right posterior frontal lobe and in parietal-occipital regions bilaterally (with microbleeding).
Beyrouti et al. (2020)	Case series	5 from a series of 6 cases (#1, #3, #4, #5, #6)	64, 85, 61, 83, 73	0/5	Cerebrovascular—Ischaemia and haemorrhage (#1), ischaemia (#3, #4, #5 and #6)	Hypertension, hypercholesterolaemia, atrial fibrillation, ischaemic heart disease, prostate cancer, stroke, chronic leg ulcers, diabetes, smoking and alcohol consumption, Gastric carcinoma and benign essential tremor	CT and MRI	UK	#1: Left inferior posterior cerebellar petechial haemorrhage and ischaemia in posteromedial temporal, occipital and thalamic territory; #3: Left temporal stem and cerebral peduncle; #4: Right striatum; #5: Right anterior-temporal and lateral temporal/perisylvian; #6: Ischaemia in the left haemi-pons and right parieto-occipital patchy pattern.
Fan et al. (2020)	Case series (from a cohort)	7 from a cohort of 86 cases with AIS	All in the age range 65-70 y.o.	2/5	Cerebrovascular— ischaemia (#1, #2, #3, #4, #5, #6) and haemorrhage (#7)	Hypertension, diabetes mellitus, coronary artery disease, ischaemic stroke, hyperlipidaemia, ischaemic stroke in the cerebellum, nasopharyngeal carcinoma, myocardial infarction developed after COVID-19 onset and COPD	CT	China	#1: Right occipital lobe and bilateral frontal and parietal lobes; #2: Left hemisphere and bilateral occipito-temporal regions; #3: Parieto-frontal regions, bilaterally; #4: Right hemisphere; #5: Left midbrain; #6: In proximity of the right periventricular tissue; #7: Sub-arachnoid space and lateral ventricles.
Saggese et al. (2020)	Single case	1	62	1/0	Cerebrovascular—ischaemia and haemorrhage	Hypertension, diabetes, previous smoker, and previous myocardial infarction	CT	Italy	Bilateral basal fronto-temporal area of ischaemia with left haemorrhagic transformation.
Chougar et al. (2020)	Single case	1	72	0/1	Cerebrovascular—ischaemia and haemorrhage	None reported	CT and MRI	France	Bilateral hypo/hyperdensities in various areas, including thalamus, basal ganglia, internal capsule, splenium of the corpus callosum, deep white matter, cerebral peduncle and pons.
Jaunmuktane et al. (2020)	Single case (from a case series)	1 out of 2 cases	#2 in her 60's	1/0	Cerebrovascular—ischaemia and haemorrhage	Hypertension	MRI	UK	Involvement of multiple brain regions, including the right thalamus, the right intraparietal sulcus, and bilateral cerebellum.
Mohamed et al. (2020)	Single case	1	Patient in her 70's	1/0	Cerebrovascular—ischaemia and haemorrhage	Severe obesity, asthma and diabetes	CT	UK	Left ischaemic infarction with areas of haemorrhage involving frontal-to-occipital territory.
Hanafi et al. (2020)	Single case	1	65	0/1	Cerebrovascular—ischaemia and haemorrhage	None reported	CT and MRI	France	Ischaemic foci in deep white matter and centrum semiovale, basal ganglia, middle cerebellar peduncle and cerebellum; haemorrhage in the globus pallidus, bilaterally.
Chen et al. (2020)	Case series	5 from a sample of 11 (#2, #3, #5, #6, #8)	81, 68, 87, 70, 89	4/1	Cerebrovascular—ischaemia (#2, #5, #6, #8) and haemorrhage (#3)	Hypertension and diabetes (none in 3 cases)	CT	China	#2: Left fronto-temporal; #3: Brainstem; #5: Pons; #6: Left parietal; #8: Basal ganglia.
Sierra-Hidalgo et al. (2020)	Case series	6 from a sample of 8 (#1, #2, #3, #4, #5, #7)	78, 83, 77, 60, 76, 61	1/5	Cerebrovascular—ischaemia (#1, #2, #3, #5) and ischaemia and haemorrhage (#4, #7)	Hypertension, diabetes, dyslipidaemia, atrial fibrillation, coronary heart disease	CT	Spain	#1: Left temporo-occipital; #2: Left fronto-temporal; #3: Left basal ganglia and fronto-temporal cortex; #4: Frontal and parietal regions, bilaterally, with right frontal haemorrhagic transformation; #5: Right posterior parietal; #7: Right cerebellum and mediotemporal, bilaterally, with haemorrhagic transformation in right mediotemporal and bilateral frontal, temporal and occipital regions.
Oliveira et al. (2020)	Single case	1	69	0/1	Cerebrovascular—vasculitis	Hypertension	MRI	Brazil	Regional vasculitis (at the level of the brainstem) with no nervous tissue involvement.
Franceschi et al. (2020)	Single case (from a case series)	1 out of 2 cases	67	0/1	Cerebrovascular—encephalopathy and haemorrhage	Hypertension, diabetes, coronary artery disease, gout and asthma	CT and MRI	USA	Oedemas in the right frontal lobe, basal ganglia, cerebellum and parieto-occipital regions, with superimposed haemorrhage in the right parieto-occipital territory.
Benameur et al. (2020)	Single case (#3 from a case series)	1	64	0/1	Encephalopathy and encephalitis	None reported	MRI	USA	Non-enhancing abnormality in the right anterior-medial temporal lobe.
Farhadian et al. (2020)	Single case	1	78	1/0	Encephalopathy and encephalitis	History of kidney transplant, on immunosuppression	MRI	USA	Atrophy and widespread periventricular and subcortical WM hyperintensities due to small vessel ischaemic disease across all lobes.
Hayashi et al. (2020)	Single case	1	75	0/1	Encephalopathy and encephalitis	Mild AD	MRI	Japan	One reversible hyperintense area in the splenium of the corpus callosum.
Abdelnour et al. (2020)	Single case	1	69	0/1	Encephalopathy, encephalitis, cerebrovascular	Hypertension, type 2 diabetes and mild chronic obstructive pulmonary disease	MRI	UK	No abnormalities apart from old infarcts in the left frontal, parietal and occipital lobes.
Mahammedi et al. (2020)	Case series	108	71 (60.5-79)^a^	39/69	Encephalopathy, encephalitis, cerebrovascular	Hypertension, diabetes, coronary artery disease, cerebrovascular disease, malignancy, MS, HIV, Behçet disease, haemoglobinopathy	CT and MRI	Italy	Neuroimaging abnormalities in 51 out of 108 cases: mostly acute ischaemic infarcts (34 out of 51), especially in the MCA territory, but in the basal ganglia in seven cases; six intracranial haemorrhages (location not specified); WM lesions in subcortical WM and the basal ganglia; rare encephalopathies in three cases and PRES in 1 case.
Paterson et al. (2020)	Case series	15 (out of 43)	60-85	3/12	Encephalopathy (#1, #2, #8), encephalitis (#12, #14, #19), cerebrovascular (#23, #24, #25, #28, #29) and PNS signs (#31, #33, #35, #38)	CADASIL, previous right occipital stroke, TIA, bladder cancer, nephrectomy, hypercholesterolemia, hypothyroidism, hysterectomy, osteoarthritis, degenerative spine disease, diabetes, hypertension, cellulitis, increased BMI, Conn Syndrome, recurrent DVT, atrial fibrillation, ischaemic heart disease, prostate cancer (Gleason Score 4+5), gastric carcinoma, benign essential tremor, cluster headache, cervical myelopathy, arrhythmia, depression, myeloma, cerebellar stroke	CT and MRI	UK	*Encephalopathies*: no abnormalities. *Encephalitis*: hyperintensities in upper pons, limbic lobes, medial thalami and subcortical cerebral WM in one case; multifocal and confluent lesions in the cerebral hemispheric WM and several microhaemorrhages in the subcortical regions in one case; multifocal lesions in periventricular WM and corpus callosum in one case. *Cerebrovascular:* Acute infarct in the right striatum and multiple cortical and subcortical microhaemorrhages in one case; acute left cerebellar and bilateral PCA infarctions in one case; subacute infarcts in frontal WM and arterial border-zones bilaterally in one case; hyperdensity due to thrombus in the left PCA and acute infarction in the left temporal stem and cerebral peduncle in one case; infarction in the right thalamus, left pons, right occipital lobe and right cerebellum in one case. *PNS signs:* no abnormalities.
Pons-Escoda et al. (2020)	Cohort	103	74 (50-90)^c^	40/63	Encephalopathy, encephalitis, cerebrovascular	Only patients with cerebrovascular accidents: hypertension, hypercholesterolemia, diabetes, smoker, atrial fibrillation	CT and MRI	Spain	No abnormalities due to COVID-19 infection in 80 patients; 23 with mainly vascular damages: one basilar strip aneurysm, one cerebellar aneurysm, three basal ganglia haematomas, one left parietal haematoma, three lobar haematomas (location not specified), one cerebellar small vessel infarction, two left prefrontal infarctions, three small vessel and eight large vessel occlusions (location not specified), one left parietal haemorrhage due to TBI.
Helms et al. (2020a)	Case series	58	63^d^	Not reported	Encephalopathy, cerebrovascular	TIA, epilepsy, MCI (in seven out of 58)	MRI (only in 13 cases)	France	Leptomeningeal enhancements in eight cases (occipito-parietal and right frontal in one case and left parietal in another case); bilateral fronto-temporal hypoperfusion in 11 cases; cerebral ischaemic stroke in three cases (right cerebellar in one case).
Helms et al. (2020b)	Cohort	140 (118 with delirium)	62 (52–70)^a^; with delirium: 62 (52–71)^a^	40/100; with delirium: 29/89	Encephalopathy, cerebrovascular	Stroke, TIA; epilepsy, MCI, migraine, TBI, aneurysm, cardiovascular diseases, haemopathies, immune diseases, diabetes, chronic liver disease, chronic renal disease, COPD, asthma, OSA	MRI (only in 32 cases with severe delirium)	France	WM microhaemorrhages across all lobes and cerebellum in seven cases and one left frontal intraparenchymal haematoma; WM hyperintensities in four cases (location not specified); subarachnoid enhancements in 17 cases (location not specified); cerebral ischaemic stroke in three cases (location not specified); hypoperfusion in 17 cases, especially in medial temporal and right frontal areas.
Krett et al. (2020)	Single case	1	69	0/1	Encephalopathy, cerebrovascular	Hypertension, diabetes, coronary artery disease	CT and MRI	Canada	CT assessment at hospital admission and after 13 days showed no abnormalities and no vasculopathy. MRI at day 13 showed diffuse multicompartmental haemorrhages (location not specified), including subarachnoid, with surrounding oedema.
Lin et al. (2020)	Cohort	278 (with CT/MRI)	71.8 (15.4)^b^	113/165	Encephalopathy, cerebrovascular	Atrial fibrillation, hypertension, hyperlipidaemia, diabetes, coronary artery disease, chronic kidney disease, COPD	CT and MRI	USA	*Encephalopathy:* PRES in three cases; Enhancements in the optic nerve in two cases and in the olfactory bulb, in the absence of volume changes, in four cases. No evidence of cortical hyperintensities, haemorrhagic encephalitis and leptomeningeal enhancements. *Cerebrovascular:* Acute and subacute cerebral infarctions in 31 cases: mainly multiterritory, but without a consistent pattern; Intracranial haematomas in 10 cases (no location specified); Microhaemorrhages in 26 cases: mainly mild and without a consistent pattern (cortical, WM, basal ganglia, cerebellum), apart from three cases with predominant damage in the corpus callosum, internal capsules, and juxtacortical WM.
Nicholson et al. (2020)	Single case (#3 from a case series)	1	62	0/1	Encephalopathy, cerebrovascular	None reported	CT and MRI	Canada	No abnormalities on CT. On MRI: enhancements in the subarachnoid and subpial spaces (no location specified); widespread hyperintensities along small cortical veins (no location specified); abnormal signal in subcortical areas, especially the corpus callosum.
Radmanesh et al. (2020b)	Case series	5 from a series of 11 cases (#3, #5, #6, #10 and #11)	60, 64, 63, 64, 62	2/3	Encephalopathy, cerebrovascular	Hypertension, diabetes, coronary artery disease, hyperlipidaemia, atrial fibrillation, obesity	MRI	USA	All cases: leukoencephalopathy in bilateral deep and subcortical WM, especially in posterior regions of temporal and occipital horns; abnormalities in precentral gyrus juxtacortical WM, centrum semiovale and corona radiata; no abnormalities in deep GM nuclei. In four cases: microhaemorrhages, mostly acute, especially in juxtacortical WM and the splenium of the corpus callosum.
**Neuropathology findings**									
Al-Dalahmah et al. (2020)	Single case	1	73	0/1	Neuropathology examination (and CT)	Hypertension, type 2 diabetes	Macroscopic and microscopic examinations	USA	*Macroscopic:* Upward herniation of the midbrain; subarachnoid haemorrhage at the base of the brain; haematoma and oedema in the right deep cerebellar WM; bilateral tonsil herniation; intra-ventricular haemorrhage and dilatation of the lateral and third ventricles; alterations in brain stem structures; cortex and cerebral WM were spared. *Microscopic:* Severe hypoxic damage to neurons in the cerebral cortex, striatum, thalamus, amygdala, hippocampus, midbrain, pontine nuclei, medullary nuclei and Purkinje cells; red blood cells and neutrophilic infiltration in the cerebellar WM; no evidence of vasculitis; microglial activation in inferior olives and dentate nuclei; inflammatory infiltrates in corpus callosum, striatum, thalamus, hippocampus, midbrain and pons, but cortex and other subcortical structures were spared; astrogliosis in OFC and SFC; inflammation in olfactory epithelium. COVID-19 present in cerebellum (including clot) and olfactory bulb, but not in the medulla oblongata.
Hernández-Fernández et al. (2020)	Case series	2 (#19 and #20, out of 23 cases)	69, 61	0/2	Neuropathology examination (and CT)	Hypertension, dyslipidaemia	Macroscopic and microscopic examinations	Spain	*Macroscopic:* In both cases: large intraparenchymal haemorrhage (one left frontal and one left parieto-temporal) with diffuse fibrin microthrombi. *Microscopic:* Disappearance of endothelial cells in arterioles, capillaries and venules; degeneration of the neuropil in the capillary periphery; local inflammation; rare inflammation of blood vessel walls; no evidence of arteriolosclerosis and cerebral amyloid angiopathy.
Jaunmuktane et al. (2020)	Case series	2	F in her 60's, M in his 50's	1/1	Neuropathology examination (and MRI)	Hypertension	Macroscopic and microscopic examinations	UK	*Macroscopic:* Bilateral pallidal infarcts, widespread acute and subacute microinfarcts and microbleeds, especially in occipital lobe WM in one case; ischaemic lesions in watershed areas in the centrum semiovale and in the right lentiform nucleus, infarcts in bilateral occipital lobe and left hippocampus and thalamus in the other case. *Microscopic:* Axonal damage but no demyelination; no evidence of microglial nodules, neuronophagia, vascular injury and vasculitis (apart from infarct areas) in either cases; inflammation in the medulla was similar to patients with other neurological diseases; leptomeningeal inflammation in right intraparietal sulcus in one case.
Bradley et al. (2020)	Case series	5 (with brain examination out of 14 cases)	57, 76, 84, 81, 42	3/2	Neuropathology examination	End-stage renal disease, type 2 diabetes, hypertension, OSA, obesity, osteoporosis, hyperlipidemia, chronic kidney disease, COPD, mitral regurgitation, complete heart block, chronic pain, arthritis, breast cancer, demyelinating neuropathy, lacunar infarcts, pneumonia, AD, anaemia	Macroscopic and microscopic examinations	USA	*Macroscopic:* Scattered subarachnoid haemorrhages in one case in one case; no abnormalities in the other four cases. *Microscopic:* Scattered subarachnoid haemorrhages and microhaemorrhages in the brainstem in one case; no abnormalities in the other four cases.
Buja et al. (2020)	Case series	3 (with brain examination out of 23 cases)	77, 42, 48	0/3	Neuropathology examination	Obesity, hypertension, splenectomy, myotonic dystrophy	Macroscopic and microscopic examinations	USA	*Macroscopic:* No abnormalities in all cases. *Microscopic:* No histopathological changes in one case (no histopathology in the other two cases).
Bulfamante et al. (2020)	Single case	1	54	0/1	Neuropathology examination	None reported	Microscopic ultrastructural examinations of ON, GR and MO	Italy	Severe and widespread damage to neurons, glia, axons and myelin sheath (ON > GR > MO); detection of viral particles compatible with COVID-19; preservation of mitochondria.
Kantonen et al. (2020)	Case series	4	63, 82, 38, 90	1/3	Neuropathology examination	Hypertension, gout, chronic kidney disease, smoking, sick sinus syndrome, coronary artery disease, myocardial infarction, peripheral artery disease, stroke, PD, type 2 diabetes, COPD, colorectal cancer, obesity, retinopathy, polyneuropathy, cellulitis, asthma, AD, osteoporosis, spinal stenosis, lung infection	Macroscopic and microscopic examinations	Finland	*Macroscopic:* Mild swelling, depigmentation of substantia nigra and locus coeruleus, enlarged perivascular spaces, microhaemorrhages in cerebral and cerebellar WM in one case; no information for the other three cases. *Microscopic:* Hypoxic injury and perivascular degeneration in all cases; WM lesions and PD pathology in one case; AD, cerebral amyloid angiopathy and Lewy bodies in one case; no evidence of COVID-19 in the neural tissue.
Matschke et al. (2020)	Case series	43	76 (70–86)^a^	16/27	Neuropathology examination	COPD, dementia, ischaemic heart disease, renal insufficiency, atrial fibrillation, cardiac insufficiency, myelofibrosis, emphysema, hypertension, diabetes, stroke, aortic aneurysm, cardiac hypertrophy, acute myeloid leukaemia, cardiomyopathy, thyroid cancer, PD, trisomy 21, epilepsy, hypoxic brain damage, cardiac arrhythmia, OSA, ulcerative colitis, lung granuloma, aortic valve replacement, hypothyroidism, lung cancer, colon cancer, paranoid schizophrenia, myelodysplastic syndrome, liver cirrhosis, dysphagia, multiple myeloma	Macroscopic and, for 23 out of 43, microscopic examinations of OB, SFC, basal ganglia (including the putamen), upper and lower medulla oblongata, cerebellar hemispheres	Germany	*Macroscopic:* No abnormalities in 13 cases; old infarctions in five cases; GM heterotopia in one case; one cerebellar metastasis from lung cancer; new infarctions in six cases (three in PCA, two in MCA, and one in ACA territories); oedema in 23, but none in 20 cases; atrophy in 20, but none in 23 cases; arteriosclerosis in all cases (mild in 12, moderate in 22, severe in 9). *Microscopic:* Astrogliosis in all cases, to variable extent, but severe in the olfactory bulb; microglia activation mainly in the olfactory bulb, medulla oblongata and cerebellum, but also in subpial and subependymal regions (sign of encephalitis); cytotoxic T cells in brain stem, frontal cortex, basal ganglia; evidence of COVID-19 in 21 patients, in the frontal lobe in nine cases (out of 23), medulla oblongata in four cases (out of eight), but also in cranial nerves.
Menter et al. (2020)	Case series	3 + 1^e^ (with brain examination out of 21 cases)	68, 96, 71	1/2	Neuropathology examination	Hypertension, atherosclerosis, obesity, MS, PD, dementia, coronary artery disease, myocardial infarction, peripheral arterial disease, infrarenal aortic aneurysm, coronary heart disease, valvulopathy, double bypass	Microscopic examinations	Switzerland	No inflammatory infiltrates or neuronal necrosis in any of the cases; mild hypoxic injury in three of the cases; hydrocephalus internus in two cases; pathological changes consistent with neurological comorbidities (MS and PD); COVID-19 presence in the brain was less prominent than in other organs, higher presence in the olfactory bulb than in the brainstem.
Reichard et al. (2020)	Single case	1	71	0/1	Neuropathology examination	ischaemic heart disease, coronary artery atherosclerosis	Macroscopic and microscopic examinations	USA	*Macroscopic:* Widespread WM haemorrhagic lesions and mild general swelling. *Microscopic:* WM haemorrhagic lesions with macrophages, axonal injuries and myelin loss, but no reactive astrogliosis; general reactive gliosis and myelin loss in WM; additional WM lesions surrounding blood vessels with macrophages, myelin loss and axonal injuries; cortical infarcts with astrogliosis; preserved subpial myelin; scattered hypoxic damage to neurons in neocortex, hippocampus (CA1), cerebellum (Purkinje cells); no infarcts in rest of the brain, basal ganglia, brainstem and spinal cord; only age-related corpora amylacea in the olfactory bulb.
Remmelink et al. (2020)	Case series	11 (with brain examination out of 17 cases)	77, 68, 64, 56, 66, 49, 63, 75, 61, 70, 53	3/8	Neuropathology examination	Coronary artery disease, cerebrovascular disease, diabetes, COPD, cancer, hypertension, chronic renal failure, liver transplant	Macroscopic and microscopic examinations	Belgium	*Macroscopic:* Recently drained subdural haematoma in one case; cerebral haemorrhage in one case. *Microscopic:* Cerebral haemorrhage or haemorrhagic suffusion in eight cases; focal ischaemic necrosis in three cases; oedema and/or vascular congestion in five cases; diffuse or focal spongiosis in 10 cases; no evidence of viral encephalitis, vasculitis, neuronal necrosis, or perivascular lymphocytic infiltration.
Youd and Moore (2020)	Case series	9 (3 positive to COVID-19, 3 likely false negatives, 3 with other respiratory infections)	88, 86, 73, 67, 33, 70, 87, 77, 68	5/4	Neuropathology examination	Type 1 an type 2 diabetes, hypertension, COPD, asthma, heart diseases, dementia, DVT, alcoholism, PD, stroke, HIV	Macroscopic examinations	UK	No abnormalities in three cases; brain atrophy in case with COVID-19 and dementia; old infarct and head injury in one case; circle of Willis atheroma in four cases.
Hanley et al. (2020)	Case series	9 (with brain analysis out of 10)	61, 64, 69, 78, 22, 24, 79, 97, 79	2/7	Neuropathology examination	COPD, ischaemic heart disease, migraine, prostatic hyperplasia, OSA, hypertension, type 2 diabetes, peripheral neuropathy, dementia, osteoarthritis, hypercholesterolaemia, trigeminal neuralgia, past bladder cancer, anaemia, glaucoma, alcohol-related liver disease, hypothyroidism, cutaneous systemic lupus erythematosus, vitamin B12 deficiency	Macroscopic and microscopic examinations on eight regions (unnamed)	UK	No necrosis was noted in any of the cases, apart from a macroscopic infarction; microglia activation and mild T cell infiltrations were observed in all the cases where these pathological features were examined (five cases); no mention of brain findings in three cases. Viral genetic material was detected in brain samples, but with variable load across cases.
Lee et al. (2021)	Case series	19	50 (5–73)^f^	4/15	Neuropathology examination	Obesity, cardiovascular disease, hypertension, type 2 diabetes, old TBI, drug use disorders	Microscopic examinations and post-mortem 11.7T MRI of OB and brainstem (in 13 cases), but also frontal cortex, basal ganglia and cerebellum in some cases.	USA	On *post-mortem* MRI: punctuate hyperintensities in nine cases, with microvascular injuries and fibrinogen leakage; punctuate hypointensities in 10 cases, with blood vessel congestion and fibrinogen leakage, but preserved vasculature; microhaemorrhages. *Microscopic:* No vascular occlusion; minimal perivascular inflammation (activated microglia, macrophage infiltrates and hypertrophic astrocytes) in 13 patients; T cells adjacent to endothelial cells in eight cases; activated microglia adjacent to neurons in five cases, suggesting neuronophagia in OB, substantia nigra, dorsal motor nucleus of the vagal nerve and the pre-Bötzinger complex. Viral genetic material was not detected in any of the brain samples.
Schurink et al. (2020)	Case series	21	68 (41–78)^f^	5/16	Neuropathology examination	Diabetes, cardiovascular disease, COPD, asthma, active solid malignancy, active haematological malignancy	Macroscopic and microscopic examinations covering all brain, spinal cord and meninges. Analysis of viral presence only in 11 cases.	The Netherlands	*Macroscopic:* most brains and meninges were normal with no atrophy, infarctions and haemorrhages. One case of pre-existing necrotising encephalopathy and one case of medial temporal atrophy due to AD. *Microscopic:* Hypoxic changes in all cases; all cases had moderate to severe microglial activation and perivascular accumulation of T cells in the most severe cases; no loss of myelin or bleeding; mild to moderate isomorphic reactive astrogliosis. Alterations were most severe in OB and medulla oblongata, but they were observed in all brain areas. COVID-19 was not detected in brain tissue in any of the cases.
Vaira et al. (2020)	Single case	1	63	1/0	Neuropathology examination	None	Biopsy of the left olfactory epithelium and MRI to investigate cause of anosmia due to COVID-19	Italy	MRI exam showed no macrostructural abnormalities in the OB. *Microscopic:* Loss of surface epithelium with no surface fibrin, inflammatory exudate, eosinophils or mast cells; minimal chronic lymphocytic inflammatory infiltrates; no abnormal neuronal infiltrates; no upregulation of the angiotensin-converting enzyme 2 receptors.

a *Median (Interquartile range)*.

b *Mean (Standard deviation)*.

c *Median (595th percentile)*.

d *Median for the whole sample, but no data for the subgroup who underwent MRI assessments*.

e *Not possible to track one case with neuropathological examination from all the materials made available*.

f *Median (range)*.

*ACA, Anterior cerebral artery; AD, Alzheimer's disease; ADEM, Acute disseminated encephalomyelitis; AIS, Acute ischaemic stroke; BMI, Body mass index; CA1, Cornu Ammonis 1; CADASIL, Cerebral autosomal dominant arteriopathy with subcortical infarcts and leukoencephalopathy; COPD, Chronic obstructive pulmonary disease; CT, Computerised tomography; DVT, Deep-vein thrombosis; FDG-PET, Fluorodeoxyglucose-positron emission tomography; GR, Gyrus rectus; MCA, Middle cerebral artery; MCI, Mild cognitive impairment; MO, Medulla oblongata; MRI, Magnetic resonance imaging; MS, Multiple sclerosis; OB, Olfactory bulb; ON, Olfactory nerve; OSA, Obstructive sleep apnoea; OFC, Orbitofrontal cortex; PCA, Posterior cerebral artery; PD, Parkinson's disease; PNS, Peripheral nervous system; PRES, posterior reversible encephalopathy syndrome; SFC, Superior frontal cortex; TBI, Traumatic brain injury; TIA, Transient ischaemic attack; WM, White matter*.

### Neuroimaging Examinations

A total of 77 manuscripts reported neuroimaging examinations of older adults aged 60 or older who tested positive for COVID-19. Studies investigated a variety of neural abnormalities associated with viral infection that fall into three main categories: encephalopathy, encephalitis and cerebrovascular injuries. Three radiological/nuclear-medicine techniques were most commonly used to monitor brain damage, especially in hospitalised patients with severe symptoms: computerised tomography (CT), magnetic resonance imaging (MRI), and fluorodeoxyglucose-positron emission tomography (FDG-PET).

#### Encephalopathy

Thirteen studies reported exclusively encephalopathy in either single cases or small case series of older patients with COVID-19. Comorbidities were not reported by all studies and were highly variable across cases, with hypertension being the most common. Other comorbidities included: history of cardiac arrest, history of lymphoma, Parkinson's disease, anorexia, depression, schizophrenia, neuropathic pain, atrial fibrillation, and epilepsy due to prior Herpes Simplex Virus-1 encephalitis.

In a case series of five patients with epileptic seizures, CT abnormalities were observed in three cases, with seizures mainly left-lateralised in frontal, parietal and temporal cortices while leukoencephalopathy was detected in the right frontal lobe and in the cerebellum bilaterally (Anand et al., 2020). Delorme et al. (2020) also reported mainly frontal alterations using MRI and FDG-PET: hypometabolism in the frontal cortex bilaterally in all four reported cases (prefrontal in three and orbitofrontal in one) and in posterior associative parieto-temporal cortices in two cases, but also hypermetabolism in the cerebellar vermis in all cases and bilaterally in the striatum in two cases. Moreover, hyperintensities were evident in the right orbitofrontal cortex in one case. Similarly, widespread hypometabolism was found in a 62-year-old man particularly in mediotemporal, brainstem, thalamic/hypothalamic, and right inferior frontal areas (Guedj et al., 2020). Hyperintensities and hypometabolism were found throughout the left hemisphere cortex, the left caudate nucleus, the thalamus, and the right cerebellum in a case with concomitant Creutzfeldt-Jakob disease (Young et al., 2020). White matter (WM) damage was observed in multiple cases with encephalopathy: deep periventricular and subcortical WM ischaemic lesions in a patient with anorexia and depression (Jang et al., 2020), and widespread WM alterations, mainly in parietal and occipital areas, in two case series (Muccioli et al., 2020; Parauda et al., 2020). Manganelli et al. (2020) found signs of gliosis in the right pons of a woman with COVID-19 using CT, but no abnormalities were found on MRI examination. Additionally, signs of inflammation of endothelial cells were observed in several cerebral arteries in a small case series where only one patient had bilateral ischaemic damage detectable on MRI (Pugin et al., 2020).

A considerable proportion of studies found either no abnormalities or old/unrelated signs of neural damage on MRI including: four single cases, two of which also included CT examinations; a patient with Miller-Fisher-like syndrome (Fernández-Domínguez et al., 2020); one with schizophrenia (Palomar-Ciria et al., 2020); and two with non-epileptic seizures (Logmin et al., 2020; Vollono et al., 2020). Moreover, no evidence of abnormalities was also found by Anand et al. (2020) on the CT scans of two out of five patients with epilepsy and by Manganelli et al. (2020) in one of the two cases investigated.

#### Encephalitis

Signs of encephalitis were investigated specifically in seven patients with COVID-19: four men (Chaumont et al., 2020; Le Guennec et al., 2020; McCuddy et al., 2020; Pilotto et al., 2020), three women (Hosseini et al., 2020; Khoo et al., 2020; Novi et al., 2020), one of whom had suspected Alzheimer's disease (AD) (Khoo et al., 2020). The majority of these studies (Chaumont et al., 2020; Khoo et al., 2020; Pilotto et al., 2020) observed no abnormalities on either MRI (all cases) or CT examinations (Pilotto et al., 2020). Diagnosis of encephalitis was variable across studies, based mainly on MRI findings and confirmed by cerebrospinal fluid abnormalities only in three studies (Chaumont et al., 2020; Novi et al., 2020; Pilotto et al., 2020). In one case encephalitis was suspected on the basis of clinical presentation and response to corticosteroid treatment (Khoo et al., 2020). However, in the case investigated by Hosseini et al. (2020), alterations of diffusion in left mediotemporal and limbic areas were found over time, on both CT and MRI scans, and these alterations were interpreted as limbic encephalitis. Le Guennec et al. (2020) found no abnormalities on CT examination, but a right-lateralised area of MRI hyperintense signal was found encompassing the orbitofrontal and medial prefrontal cortices and the caudate nucleus, that gradually resolved over one month. Similarly, hyperintensities with restricted diffusion were observed in deep WM, the corpus callosum and the left brachium pontis in a patient, with clinical improvement over a period of 8 days (McCuddy et al., 2020). Another case presented with multiple gadolinium-enhancing lesions affecting the spinal cord, the optic tract, temporal, occipital and frontal areas suggesting acute disseminated encephalomyelitis (Novi et al., 2020).

#### Cerebrovascular Events

Forty-five studies in total, mostly single-case or case-series reports, described the topological features of brain involvement due to acute cerebrovascular events occurring concomitantly with COVID-19 infection.

The only group study (Radmanesh et al., 2020a) included a total of 242 adults (68.7 ± 16.7 years old) and was based on recruitment carried out in a single academic clinical centre. The most common finding in this cohort was the presence of acute/sub-acute infarcts (~19.4% of patients), followed by radiological evidence of abnormal microangiopathy (~11%), intracranial COVID-19-related haemorrhage (~3%), and in one patient there was an anoxic injury due to supra- and infra-tentorial haemorrhage. Additional details were provided for the following six patients: a 74-year-old man with a right inferior frontal haemorrhage, a 61-year-old woman with a left parietal haemorrhage, a 62-year-old man with a left frontal ischaemic stroke, a 77-year-old woman with a large right fronto-temporal ischaemia, a 63-year-old man with an acute infarct in the left cerebellum and a 78-year-old man with ischaemic involvement of the middle cingulate and the body of the corpus callosum. The map of neural damage for the remaining patients was not described.

When single cases and case series were assessed, a total of 120 patients met the demographic and methodological criteria set by this review study. These clinical reports included 84 cases of ischaemic stroke, 23 cases of haemorrhagic events, 10 patients with significant mixed ischaemic and haemorrhagic processes, two patients with a form of encephalopathy and microhaemorrhages and one case of vasculitis without any noticeable involvement of brain tissue.

##### Ischaemia

Various vascular findings (including coagulopathy and cardioembolism, with vessel occlusion, thrombosis, or stenosis) were responsible for the ischaemic events described in the literature, and the territory affected by the CT/MRI-informed changes involved multiple neural structures. All cerebral lobes have been reported to be affected by ischaemic events associated with COVID-19 including the frontal lobe (Avula et al., 2020; Basi et al., 2020; Fan et al., 2020; Hernández-Fernández et al., 2020; Jillella et al., 2020; Katz et al., 2020; Mohamud et al., 2020; Morassi et al., 2020; Papi et al., 2020; Tiwari et al., 2020; Zayet et al., 2020; Zhang et al., 2020) with additional involvement of pericentral areas (Mohamud et al., 2020; Morassi et al., 2020), the temporal lobe (Beyrouti et al., 2020; Jillella et al., 2020; Mohamud et al., 2020; Morassi et al., 2020; Papi et al., 2020; Tiwari et al., 2020; Zhai et al., 2020; Zhang et al., 2020), fronto-temporal regions (Barrios-López et al., 2020; Chen et al., 2020; Hernández-Fernández et al., 2020; Mohamud et al., 2020; Papi et al., 2020; Sierra-Hidalgo et al., 2020), the parietal lobe (Bolaji et al., 2020; Chen et al., 2020; Fan et al., 2020; Hernández-Fernández et al., 2020; Jillella et al., 2020; Papi et al., 2020; Sierra-Hidalgo et al., 2020; Zayet et al., 2020; Zhang et al., 2020), fronto-parietal regions (Fan et al., 2020; Goldberg et al., 2020; Jillella et al., 2020; Mohamud et al., 2020; Morassi et al., 2020; Tunç et al., 2020; Viguier et al., 2020; Zayet et al., 2020; Zhang et al., 2020), the occipital lobe (Diaz-Segarra et al., 2020; Fan et al., 2020; Hernández-Fernández et al., 2020; Janjua and Moscote-Salazar, 2020; Jillella et al., 2020; Morassi et al., 2020; Zhang et al., 2020), and the parieto-occipital (Avula et al., 2020; Beyrouti et al., 2020; Jillella et al., 2020; Tiwari et al., 2020; Zayet et al., 2020), temporo-parietal (Jillella et al., 2020), or temporo-occipital territory (Barrios-López et al., 2020; Fan et al., 2020; Sierra-Hidalgo et al., 2020). Seven of the 84 cases with cerebral ischaemia did show a cerebral involvement but no detailed description was provided to map brain damage with accuracy (Barrios-López et al., 2020; Co et al., 2020; Diaz-Segarra et al., 2020; Fan et al., 2020; Hanafi et al., 2020; Kananeh et al., 2020; Tiwari et al., 2020; Tunç et al., 2020; Zhang et al., 2020). Additionally, a number of studies have documented an involvement of the insular region (Hernández-Fernández et al., 2020; Jillella et al., 2020; Papi et al., 2020; Zhai et al., 2020), of limbic regions located in the mediotemporal lobe (Avula et al., 2020; Zhai et al., 2020) and in the cingulate gyrus (Morassi et al., 2020), and of the dorsal striatum (Beyrouti et al., 2020; Hanafi et al., 2020; Mohamud et al., 2020; Morassi et al., 2020; Tunç et al., 2020) or, more generally, of the basal-ganglia territory (Chen et al., 2020; Janjua and Moscote-Salazar, 2020; Jillella et al., 2020; Sierra-Hidalgo et al., 2020; Tiwari et al., 2020; Zhang et al., 2020). Two patients presented with an infarction affecting the corpus callosum (Sparr and Bieri, 2020). Other than the cerebrum, evidence of diencephalic ischaemia affecting the thalamus has been reported in six patients (Hernández-Fernández et al., 2020; Jillella et al., 2020; Morassi et al., 2020; Tiwari et al., 2020), and cerebellar involvement in 11 cases (Barrios-López et al., 2020; Basi et al., 2020; Hanafi et al., 2020; Hernández-Fernández et al., 2020; Janjua and Moscote-Salazar, 2020; Jillella et al., 2020; Morassi et al., 2020; Sierra-Hidalgo et al., 2020; Sparr and Bieri, 2020; Zayet et al., 2020). Brainstem infarction was described in six patients: three in the pons (Beyrouti et al., 2020; Chen et al., 2020; Tunç et al., 2020); one in the midbrain (Fan et al., 2020); one in the cerebral peduncle (Beyrouti et al., 2020); and one in an unspecified brainstem area (Zhang et al., 2020). Two cases of cerebrovascular occlusion with no acute neural damage were described by Mohamud et al. (2020).

##### Haemorrhage

A heterogeneous pattern was also observed in the case series with a pure haemorrhagic presentation (without any concurrent significant ischaemic or encephalopathic features). In the 11 cases presenting with a large haemorrhage, the regions involved were the left temporal lobe (Ghani et al., 2020; Hernández-Fernández et al., 2020; Sharifi-Razavi et al., 2020), the right temporal/insular territory (Benger et al., 2020), the temporal lobe bilaterally (Roy-Gash et al., 2020), the left frontal lobe (Hernández-Fernández et al., 2020), the parietal lobe bilaterally (Sabayan et al., 2021), the cerebellum (Al-Dalahmah et al., 2020), the basal ganglia (Pavlov et al., 2020), and a large portion of the right hemisphere (Keaney and Mumtaz, 2020). In one case, the regions affected included the lateral ventricles and the subarachnoid space with no additional details reported (Fan et al., 2020). In the case of a patient, a frontal haemorrhage was due to the rupture of an aneurysm (Muhammad et al., 2020). Eight patients, finally, showed evidence of subcortical white-matter microbleeds with the involvement of the brainstem (Chen et al., 2020) and of the corpus callosum (Fitsiori et al., 2020), and of these latter, four also presented with a mixed pattern of widespread microbleeds and lacunar haemorrhagic infarcts.

##### Mixed Ischaemia and Haemorrhage Pattern

Of the cases with a mixed ischaemic-haemorrhagic presentation, one patient showed evidence of right frontal subarachnoid bleeding, left intraparenchymal hematoma, and a concurrent pattern of confluent hyperintensities affecting parieto-occipital regions bilaterally (Hernández-Fernández et al., 2020). A second patient presented with cortical/sub-cortical haemorrhage in the temporal and occipital lobe, multiple sub-arachnoid haemorrhages and bilateral parieto-occipital hyperintensities (Hernández-Fernández et al., 2020). A third patient suffered from ischaemia with haemorrhagic transformation in left temporo-parietal regions (Saggese et al., 2020). A fourth patient showed haemorrhage in proximity of the left cerebellar hemisphere and concurrent ischaemic changes in occipital, thalamic and posteromedial territories (Beyrouti et al., 2020). A fifth patient showed a subcortical ischaemic event affecting the thalamus, basal ganglia, internal capsule and the splenium, with concomitant haemorrhage in the right cerebral peduncle and pons (Chougar et al., 2020). A sixth patient showed numerous hyperintensities, leukoaraiosis in the right intraparietal sulcus and microhaemorrhages in the left centrum semiovale, thalamus, left cerebellum and left anterior temporal lobe (Jaunmuktane et al., 2020). A seventh patient presented with bilateral ischaemic-haemorrhagic infarctions affecting, above all, a large proportion of the left hemisphere from frontal to occipital regions (Mohamed et al., 2020). An eight patient had several ischaemic regions scattered across his white matter including the cerebellum, deep white matter and centri semiovale, with a concomitant lenticular haemorrhage (Hanafi et al., 2020). A ninth patient showed ischaemic changes affecting frontal and parietal regions, bilaterally, with haemorrhagic transformation in the right frontal lobe (Sierra-Hidalgo et al., 2020). A tenth patient presented with multiple infarctions in regions such as the medial temporal lobe and cerebellum, and showed concurrent bilateral haemorrhages in frontal, temporal and occipital territories, and also in the right medial temporal lobe (Sierra-Hidalgo et al., 2020).

##### Vasculitis

One patient presented with systemic vasculitis but no changes to the nervous tissue were reported (Oliveira et al., 2020).

#### Multiple Findings

Twelve studies used neuroimaging to investigate a multiplicity of different types of neural damage. Studies on five single cases reported a range of different findings, most consistently involving WM damage. A non-enhancing abnormality in the right anterior-medial temporal lobe was noted by Benameur et al. (2020). Atrophy and widespread periventricular and subcortical WM ischaemic lesions were found in a 78-year-old woman (Farhadian et al., 2020). Both studies investigated inflammatory changes compatible with encephalitis, by means of neuroimaging and cerebrospinal fluid (CSF) analysis, although the relationship with COVID-19 infection remained unclear. One reversible WM hyperintensity due to encephalopathy/encephalitis (diagnosed on the basis of MRI findings only) was found in the splenium of the corpus callosum in one patient with mild AD (Hayashi et al., 2020). One study (Nicholson et al., 2020), instead, found multiple abnormalities only on MRI, but not on CT scans, spreading from subarachnoid and subpial spaces (enhancements) to areas of hyperintense signal in perivascular regions and in subcortical WM, especially across the corpus callosum. Similarly, Krett et al. (2020) observed diffuse haemorrhages on MRI, in the absence of vasculopathy, across multiple brain compartments, including the subarachnoid space. Finally, two patients were reported having posterior reversible encephalopathy syndrome: the first one presented with hyperintensities (but no evidence of stenosis) in the right posterior frontal lobe, in the left centrum semiovale and in parieto-occipital regions bilaterally, accompanied by microbleeds in this latter territory (Hernández-Fernández et al., 2020); while the second one showed oedema extending to parieto-occipital regions bilaterally, cerebellum, right frontal lobe and basal ganglia, with evidence of an haemorrhagic process in left parieto-occipital areas (Franceschi et al., 2020).

Case series and cohort studies included patients with a variety of comorbidities, especially cardiovascular pathologies such as hypertension, history of stroke and transient ischaemic attack, atrial fibrillation and deep-vein thrombosis. However, multiple cases with diabetes, a history of cancer, mild cognitive impairment, chronic obstructive pulmonary disease and kidney pathologies were reported. Paterson et al. (2020) investigated patients falling into four main categories, depending on the predominant type of neural damage found: encephalopathy, encephalitis, cerebrovascular involvement, and peripheral nervous system signs. No brain abnormalities were reported in those with encephalopathy and peripheral nervous dysfunctions. Three patients with encephalitis, defined by means of both MRI and cerebrospinal fluid assessments, showed different pathological changes: hyperintense areas in the pons, limbic areas, medial thalamic nuclei, and subcortical cerebral WM were detected in one patient, while in the other two cases different types of subcortical WM lesions were mainly observed. Great variability in the type of cerebrovascular injuries and in the brain areas affected was also observed in five cases, since haemorrhages and infarcts were detected mainly in cerebellar/brainstem areas, but also in cerebral WM (frontal and occipital) and in the basal ganglia. Thirteen out of 58 cases reviewed by Helms et al. (2020a) showed different cerebral abnormalities, yet almost all patients (11 out of 13) presented with bilateral fronto-temporal hypoperfusion detected with arterial spin labelling MRI. The same research group also found a similar pattern of hypoperfusion, mainly in mediotemporal and right frontal areas, in 17 out of 32 patients with COVID-19 who presented with severe delirium (Helms et al., 2020b). Moreover, WM microhaemorrhages were noted across all cerebral lobes and the cerebellum in seven cases and a left frontal intraparenchymal haematoma was detected in one case. Radmanesh et al. (2020b), instead, observed in five cases that WM damage, both as leukoencephalopathy and microhaemorrhages, was especially present in posterior occipital and temporal areas, in the corpus callosum, centrum semiovale, corona radiata, and in juxtacortical WM in the precentral gyrus, while deep grey matter nuclei were spared.

A cohort study (Pons-Escoda et al., 2020) found that only 23 out of 103 patients with COVID-19 presented with cerebrovascular accidents, mainly located in the basal ganglia (three cases), prefrontal (two cases), parietal, and cerebellar (one case each) regions. However, the location of some of the cerebrovascular injuries (three lobar haematomas) was not included. No cases of encephalitis were detected by neuroimaging examinations. Similarly, the largest cohort including 278 patients assessed with either CT or MRI (Lin et al., 2020) found little evidence of encephalopathy due to COVID-19: posterior reversible encephalopathy syndrome was present in three cases, while areas of signal enhancement in the optic nerve were present in two cases and in the olfactory bulb (with no evidence of volume changes) in four cases. However, cerebrovascular events were reported to be more common: infarctions were present in 31 cases, mainly in multiple vascular territories and without a consistent pattern across patients. Microhaemorrhages (26 cases) were mild and without a consistent pattern in the overall sample, but in three patients lesions were predominantly localised in the corpus callosum, in both internal capsules and in juxtacortical WM. Similarly, Mahammedi et al. (2020) observed neuroimaging abnormalities, especially of cardiovascular origin, in 47% of 108 hospitalised patients with COVID-19 presenting with neurological symptoms. Ischaemic infarcts represented the most common finding observed in various vascular territories, but also in the basal ganglia, with WM damage found in subcortical and basal ganglia areas. Encephalopathy was rare and only one case of posterior reversible encephalopathy was reported in this series.

A few more studies observed no recent and acute neural changes that could be ascribed to COVID-19 infection: Abdelnour et al. (2020) reported the case of a man who presented only with old infarcts and no signs of encephalitis on MRI, no brain abnormalities were found in 80 out of 103 patients by Pons-Escoda et al. (2020) and two studies detected either no signs of encephalopathy (Paterson et al., 2020) or no cortical hyperintensities, haemorrhagic encephalitis and leptomeningeal enhancement in all patients included (Lin et al., 2020).

#### Cognitive Correlates of Neuroimaging Findings

A subset of neuroimaging studies carried out in older patients also reported details of cognitive symptoms, although the relationship between neural damage and cognitive deficits was rarely discussed and not always transparent. For example, delirium was not associated with specific neuroimaging findings: Helms et al. (2020b) observed this symptom in people with WM damage, fronto-temporal hypoperfusion, stroke, and haematomas, while other patients with delirium had no MRI abnormalities at all (Paterson et al., 2020). Decline in, or loss of, consciousness was also reported in patients with right frontal ischaemia (Basi et al., 2020), right temporal haemorrhage (Sharifi-Razavi et al., 2020), lesions of the left midbrain (Fan et al., 2020) and of the left ventromedial prefrontal cortex (Muhammad et al., 2020), extensive right-sided (Fan et al., 2020) or left-sided lesions (Mohamed et al., 2020), diffuse WM lesions (McCuddy et al., 2020; Muccioli et al., 2020), diffuse cerebrovascular alterations (Pugin et al., 2020) and also in the absence of MRI abnormalities (Manganelli et al., 2020; Mohamud et al., 2020). Similarly, altered mental status was observed in patients with one lesion in the splenium (Sparr and Bieri, 2020), scattered WM lesions (Farhadian et al., 2020), and microbleeds (Fitsiori et al., 2020), in a case with haemorrhage in the right parieto-occipital territory (Franceschi et al., 2020), but in most cases alterations in mental state were not associated with any specific MRI finding (Radmanesh et al., 2020a,b).

A few cases were also described of patients presenting with some degree of unspecified cognitive decline, present either at hospital admission or developing during hospitalisation, that was associated with multiple haemorrhages in one case (Krett et al., 2020) and no structural neuroimaging findings in other two cases (Khoo et al., 2020; Pilotto et al., 2020). More specific cognitive symptoms were also observed: executive dysfunction in patients with frontal hypometabolism (Delorme et al., 2020), memory and attention deficits associated with persistent delirium in a patient with diffuse ischaemic damage mainly in temporal and limbic areas (Hosseini et al., 2020), left-sided neglect due to right frontal ischaemia (Avula et al., 2020) and aphasia in cases of diffuse left-sided (Beyrouti et al., 2020), left frontal (Jillella et al., 2020), and bilateral cerebrovascular injuries, mainly in temporal areas (Jillella et al., 2020; Roy-Gash et al., 2020; Saggese et al., 2020).

### Neuropathological Examinations

Sixteen studies reported various macroscopic and microscopic results of neuropathological examinations: four single cases, three *post-mortem* examinations (Al-Dalahmah et al., 2020; Bulfamante et al., 2020; Reichard et al., 2020) and one *ante-mortem* biopsy of the olfactory epithelium (Vaira et al., 2020), and 12 case series (Bradley et al., 2020; Buja et al., 2020; Hanley et al., 2020; Hernández-Fernández et al., 2020; Jaunmuktane et al., 2020; Kantonen et al., 2020; Matschke et al., 2020; Menter et al., 2020; Remmelink et al., 2020; Schurink et al., 2020; Youd and Moore, 2020; Lee et al., 2021), for a total of 132 patients who died with COVID-19 (65 of whom aged 60 or older). Many comorbidities were reported in 14 out of the 16 studies, especially: hypertension, diabetes, kidney diseases and a range of cardiovascular pathologies. A few patients were also affected by other neurodegenerative conditions, such as AD, Parkinson's disease and multiple sclerosis.

#### Macroscopic Findings

**Figure 2:**
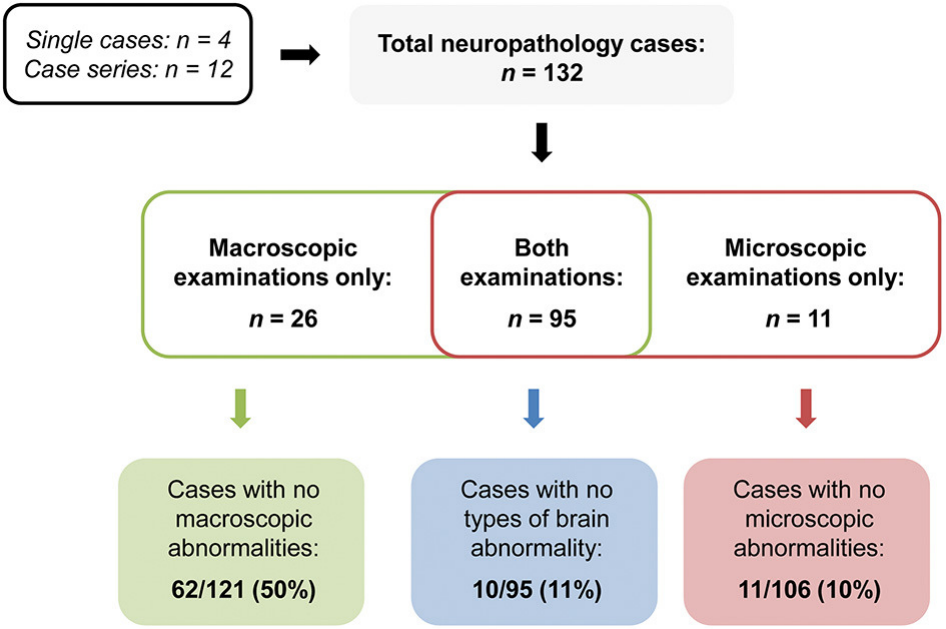
Summary of the rates of null neuropathological findings.

All but two studies (Bulfamante et al., 2020; Menter et al., 2020) reported the results of macroscopic inspections of patients' brains. The majority of the papers observed cerebrovascular damage of different type. Haemorrhages were found in seven patients and damage was located in: the right cerebellum (Al-Dalahmah et al., 2020), left frontal and left parieto-temporal lobes (Hernández-Fernández et al., 2020), subarachnoid space (Bradley et al., 2020), and both cerebral and cerebellar WM (Kantonen et al., 2020; Reichard et al., 2020). Cerebrovascular damage either without a specific localisation or widespread throughout the brain was reported by three studies (Hanley et al., 2020; Remmelink et al., 2020; Lee et al., 2021). New infarctions were found in eight cases in: bilateral globus pallidum, occipital lobe WM and left hippocampus and thalamus (Jaunmuktane et al., 2020); and in territories of the posterior (three cases), middle (two cases), and anterior (one case) cerebral arteries (Matschke et al., 2020). Ischaemic lesions were noted in the centrum semiovale and in the right lentiform nucleus in one case (Jaunmuktane et al., 2020). Matschke et al. (2020) reported non-specific oedema in 23 cases, atrophy in 20 cases and arteriosclerosis in all 43 cases. Additionally, tentorial and foramen magnum herniations were found in one case (Al-Dalahmah et al., 2020), while depigmentation of the substantia nigra and locus coeruleus, and enlarged perivascular spaces were noted in another case (Kantonen et al., 2020). However, no evidence of macroscopic brain abnormalities was observed in 50% of the neuropathological cases (Bradley et al., 2020; Buja et al., 2020; Hanley et al., 2020; Matschke et al., 2020; Remmelink et al., 2020; Schurink et al., 2020; Vaira et al., 2020; Youd and Moore, 2020) (Figure 2).

#### Microscopic Findings

All but one study (Youd and Moore, 2020) carried out microscopic pathological analyses on samples of neural tissue. Null findings were observed in the microscopic examination of only 11 cases of older adults deceased with COVID-19 included in three studies (Bradley et al., 2020; Buja et al., 2020; Hanley et al., 2020) (Figure 2).

Damage was observed in a wide variety of neural cells. Hypoxic damage was found in a single case in neurons across the cerebral cortex, striatum, thalamus, amygdala, hippocampus, midbrain, pontine nuclei, medullary nuclei, and Purkinje cells (Al-Dalahmah et al., 2020). Non-specific hypoxic damage was also reported by other studies (Kantonen et al., 2020; Menter et al., 2020; Schurink et al., 2020). WM axonal loss and demyelination were detected in sites of vascular damage in combination with scattered hypoxic damage to neurons in neocortex, hippocampus (CA1) and the cerebellum in one case (Reichard et al., 2020). Moreover, non-specific WM axonal damage in the absence of demyelination (Jaunmuktane et al., 2020) and WM lesions (Kantonen et al., 2020) were also reported. In one patient, severe damage to neurons, glia, axons and myelin sheath was found to be more prominent in the olfactory nerve, followed by the gyrus rectus and the medulla oblongata (Bulfamante et al., 2020).

Signs of inflammation were also found throughout the central nervous system (CNS) in the corpus callosum, striatum, thalamus, hippocampus, midbrain and pons, and olfactory epithelium of one patient (Al-Dalahmah et al., 2020), leptomeningeal inflammation in the right intraparietal sulcus in one case (Jaunmuktane et al., 2020), and widespread across the brainstem, the frontal cortex and the basal ganglia in a case series (Matschke et al., 2020). In particular, microglial activation was reported by several studies (Hanley et al., 2020; Schurink et al., 2020; Lee et al., 2021) and across different regions, namely: the inferior olives and dentate nuclei (Al-Dalahmah et al., 2020), the medulla oblongata, cerebellum, olfactory bulb, and subpial and subependymal regions (Matschke et al., 2020). Additionally, astrogliosis was found in all cases analysed by Matschke et al. (2020), especially in the olfactory bulb, and in the orbitofrontal and superior frontal cortices of a patient examined by Al-Dalahmah et al. (2020). A few studies, instead, found no traces of either increased microglia activation (Jaunmuktane et al., 2020), vasculitis (Al-Dalahmah et al., 2020; Jaunmuktane et al., 2020; Remmelink et al., 2020), which was reported to a mild extent only by one neuropathological study (Hernández-Fernández et al., 2020), or of any inflammatory processes (Menter et al., 2020; Remmelink et al., 2020).

A variety of cerebrovascular injuries, often reported as a general finding without brain localisation (Remmelink et al., 2020), was observed in endothelial cells in arterioles, capillaries, and venules and degeneration of the pericapillary neuropil (Hernández-Fernández et al., 2020). Subarachnoid haemorrhages and microhaemorrhages in the brainstem were reported in one patient (Bradley et al., 2020) and a haemorrhagic WM lesion in one case (Reichard et al., 2020). The absence of cerebrovascular damage was recorded by one neuropathological study (Jaunmuktane et al., 2020).

One study that investigated the olfactory epithelium of a patient with COVID-19 and anosmia found a reduction in surface with minimal levels of chronic lymphocytic inflammatory infiltrates (Vaira et al., 2020). MRI examination revealed no macrostructural abnormalities in the olfactory bulb.

**Figure 3:**
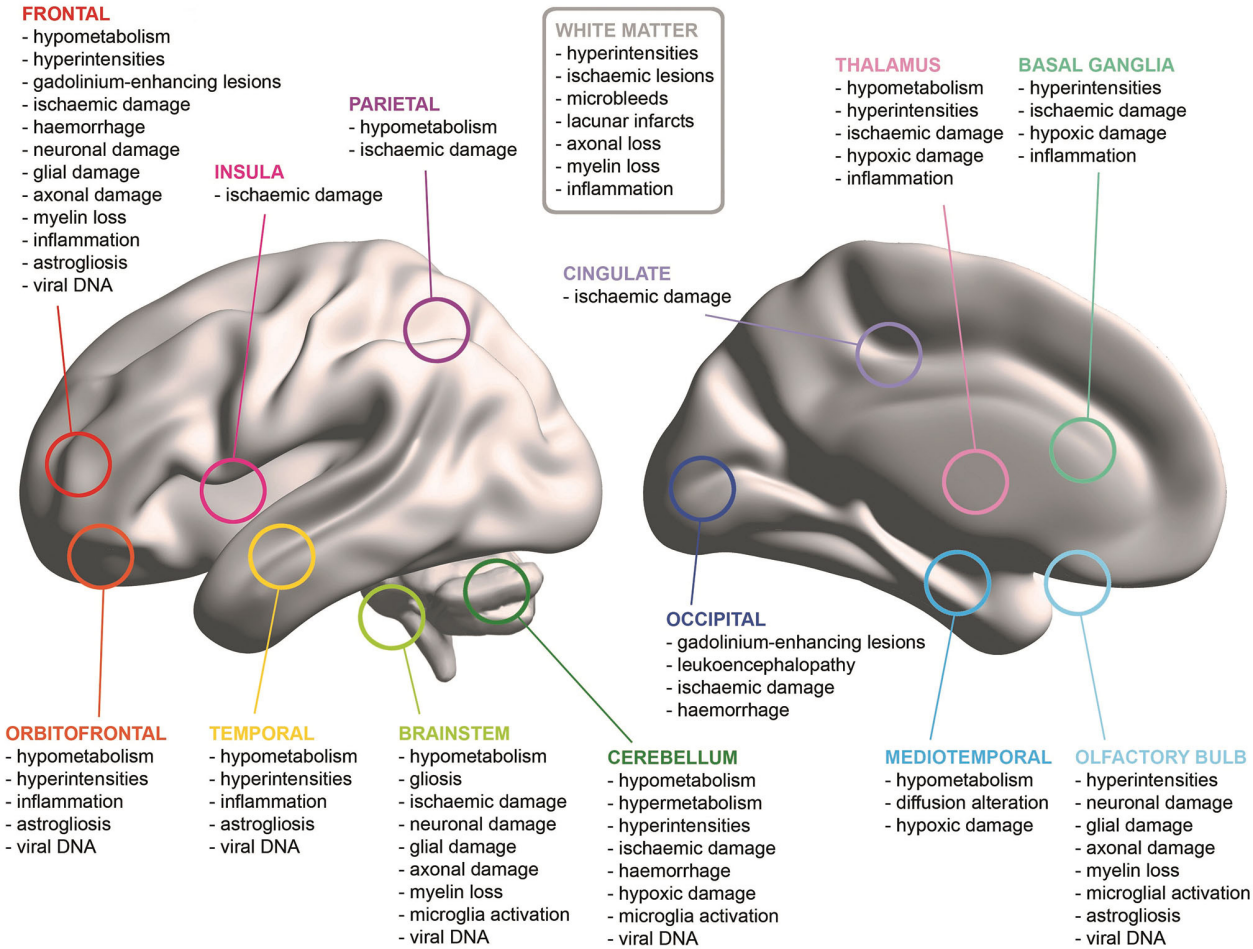
Summary of the most common neural findings across the brain.

Finally, a few studies also investigated the presence of COVID-19 in the CNS tissue samples. Although three studies (Kantonen et al., 2020; Schurink et al., 2020; Lee et al., 2021) found no evidence of viral infection in the CNS, this was observed repeatedly across different brain regions: the olfactory nerve (Al-Dalahmah et al., 2020; Bulfamante et al., 2020; Matschke et al., 2020; Menter et al., 2020), frontal lobe (Bulfamante et al., 2020; Matschke et al., 2020), and brainstem (Bulfamante et al., 2020; Matschke et al., 2020; Menter et al., 2020), especially in the medulla oblongata, and in the cerebellum of a patient with cerebellar haemorrhage (Al-Dalahmah et al., 2020) (Figure 3). Hanley et al. (2020) detected the presence of COVID-19 across the brain, but viral load was highly variable across cases.

Only a minority of the neuropathological investigations assessed whether microscopic alterations and viral presence in the neural tissue was associated with neuroimaging and clinical findings. Pervasive vascular damage due to haemorrhages, as expected, was consistently detected by both macroscopic and microscopic examinations (Al-Dalahmah et al., 2020; Hernández-Fernández et al., 2020) and linked to the presence of viral genetic material in the brainstem in one patient, possibly due to blood contamination (Al-Dalahmah et al., 2020). Anosmic patients with COVID-19 presented with alterations in both the olfactory epithelium (Vaira et al., 2020) and bulb (Bulfamante et al., 2020); however, such microstructural alterations did not correlate with volumetric changes in the olfactory bulb as assessed by means of MRI (Vaira et al., 2020). Similarly, patients who presented with delirium and altered mental status before death showed no specific neuropathological signatures (Kantonen et al., 2020; Schurink et al., 2020; Lee et al., 2021) and some had no CNS tissue abnormalities at all (Bradley et al., 2020).

## Discussion

As of December 2020, the COVID-19 pandemic has become established across the planet for at least 12 months. The medical community has promptly responded to the emergency to the best of their capabilities and has documented the mechanistic and clinical features of this viral infection, putting emphasis on the nervous system as one of its major targets. It is thus particularly important for cognitive and clinical neuroscientists to study the link between the neural effects of COVID-19 and changes in cognitive and psychiatric/behavioural functioning. At present, however, the evidence available in the literature is limited to (1) the acute effects of the virus and (2) patients who have contracted the infection and developed symptoms of sufficient concern to justify hospitalisation and radiological investigations. As a result, we have only partial knowledge of the link between COVID-19 infection and mental abilities. Although characterisation of the long-term neural effects of COVID-19 infection will be investigated in due course, and indeed there are several ongoing studies at present, it is of primary importance to review the current evidence in order to define a theoretical backbone in support of future experimental studies.

At first glance, the literature currently available on the neural changes observed in older adults with COVID-19 shows great variability of findings across studies. While variability is expected in any condition (in this case, especially when the damage route is *via* cerebrovascular and inflammatory mechanisms of typical patchy presentations), it is possible that a better defined pattern will be apparent in the long term. Many examples of brain damage of vascular aetiology associated with COVID-19 infection were noted, thus suggesting that the cerebrovascular system may be particular susceptible. In general, neuroimaging findings do not appear to be distinctly associated with a set of specific brain areas (Jang et al., 2020; Krett et al., 2020; Mahammedi et al., 2020) and a variety of neural changes have been observed everywhere in the brain, the meninges and the cerebrovascular system (Lin et al., 2020; Pons-Escoda et al., 2020) (Figure 3). However, some recurrent findings emerged from the studies focussing on encephalopathies. In particular, multiple papers reported WM lesions of variable aetiology, presentation, and location (Anand et al., 2020; Benameur et al., 2020; Farhadian et al., 2020; Hayashi et al., 2020; Jang et al., 2020; Nicholson et al., 2020) that have also been commonly observed in larger cohorts (Paterson et al., 2020; Radmanesh et al., 2020b). A considerable amount of older adults with COVID-19 examined with FDG-PET and arterial spin labelling MRI presented with hypoperfusion in bilateral frontal and temporal cortices (Delorme et al., 2020; Guedj et al., 2020; Helms et al., 2020a,b), while both hyperperfusion and hypoperfusion have been observed in the cerebellum (Delorme et al., 2020; Young et al., 2020). It must be noted, however, that, encephalopathy, is an umbrella term and that different pathophysiological changes, some primarily related to COVID-19 infection and some related to collateral events, might have contributed to such condition. While accounts of patients with encephalopathy responsive to corticosteroids suggest an immune-mediated pathogenesis (Pilotto et al., 2020; Pugin et al., 2020), intubation and mechanical ventilation might have also contributed to neural damage in a minority of patients (Delorme et al., 2020; Parauda et al., 2020). Indeed, a few patients with diffuse subcortical damage developed encephalopathy after sedation (Muccioli et al., 2020) and extubation (Lin et al., 2020). Therefore, it cannot be excluded that invasive medical procedures as well as several pre-existing comorbidities in older patients might have contributed to the heterogeneity of neuroimaging findings.

In several cases, brain abnormalities of any type were detected bilaterally across most cerebral and cerebellar regions. Cerebrovascular damage, instead, was most frequently lateralised to a single hemisphere at the individual level, although both brain sides appeared to be equally affected, overall. A trend for a higher rate of cerebrovascular findings on the right side of the brain was observed across all lobes, but, since no studies have investigated whether one of the two hemispheres may be more prone to COVID-19-related damage, any speculation on this issue appears premature. However, vascular injuries of any type appeared to be more often located in the frontal lobes, followed by parietal, temporal and occipital areas. This pattern appears to be similar to that detected in other critical illnesses, e.g., sepsis is associated with dysfunction in cerebrovascular regulation and, consequently, hypoperfusion, particularly in mediotemporal and frontal areas, is frequently observed (Tauber et al., 2021). Moreover, a case series of three patients with Middle East respiratory syndrome coronavirus showed similar widespread bilateral brain abnormalities in frontal, temporal as well as subcortical areas on MRI assessment (Arabi et al., 2015). A study that compared the clinical profiles of patients with COVID-19 and patients with Influenza virus revealed that the flu virus was associated with a lower risk of developing an ischaemic stroke (Merkler et al., 2020). No information on the anatomical localisation of these acute vascular events was provided in this study, however.

In a minority of cases, several cognitive alterations were reported. Delirium, loss of consciousness and altered mental status appeared not to be associated with specific pathological signatures, possibly because such symptoms are vaguely defined and, therefore, may arise in patients because of different medical and environmental conditions. However, they could also be caused mainly by functional, rather than structural, cerebral alterations that have not been investigated by the majority of the studies currently available. Indeed, hypoperfusion of medial temporal and right frontal areas was observed to be pronounced in patients who presented with severe delirium (Helms et al., 2020b). In contrast, more specific impairments were observed in cases with injuries of differing aetiologies to brain structures known to be involved in the functions affected: executive function decline in patients with frontal hypometabolism (Delorme et al., 2020), memory impairment due to limbic damage (Hosseini et al., 2020), neglect in a case with right frontal damage (Avula et al., 2020), and aphasia due to left-sided and temporal injuries (Beyrouti et al., 2020; Jillella et al., 2020; Roy-Gash et al., 2020; Saggese et al., 2020).

Although longitudinal investigations on neural and cognitive alterations are not available yet, these findings are particularly relevant for the long-term cognitive health of older patients. Indeed, signs of hypoperfusion in frontal and temporal lobes were consistently highlighted across studies; these brain regions mainly consist of associative cortex, implicated in memory, executive functions as well as complex behavioural control (Badre and Nee, 2018; Jackson et al., 2018). Such negative consequences of COVID-19 on the neural tissue in these areas may either be transient or a driver for long-lasting effects on mental and cognitive health of patients. The increased frequency of acute ischaemic strokes following COVID-19 infections in comparison to other respiratory conditions might also reduce brain reserve in older individuals. These negative neural consequences, in turn, might increase the risk of developing a variety of neurodegenerative conditions leading to dementia, e.g., sporadic AD or fronto-temporal lobar degeneration (Maillet and Rajah, 2013; Mann and Snowden, 2017), or might accelerate the clinical manifestation of existing latent sub-clinical conditions. The possibility exists that significant CNS involvement in COVID-19 infection may join other vascular components of “brain-at-risk” for cognitive decline alongside mid-life hypertension, diabetes, smoking, and many other reported factors. This might be particularly evident in carriers of the ε4 variant of the apolipoprotein E (ApoE) gene, the most strongly established genetic risk for sporadic AD that also modulates cardiovascular diseases and cellular processes related to viral infections (Finch and Kulminski, 2020). ApoE ε4 appears to be a risk factor common to both AD and COVID-19-related outcomes, since symptom severity (Kuo et al., 2020a) and mortality rates (Kuo et al., 2020b) have been found to be significantly worse in ε4 homozygotes, independently of any common comorbidities (i.e., coronary heart disease, dementia, diabetes, and hypertension). Therefore, longitudinal monitoring of older adults who have recovered from COVID-19 infection, especially those with known genetic vulnerabilities, should be taken into consideration not only to ascertain the long-term impact of COVID-19 on the central nervous system, but also to detect any signs of cognitive decline early and arrange a prompt management plan. In fact, one study on young patients who have been assessed with MRI 3 months after recovery from COVID-19 found increased volumes in olfactory, cingulate and both medial and lateral temporal cortices that correlated negatively with loss of olfactory and memory functions (Lu et al., 2020), thus suggesting a compensatory role of these hypertrophic neurovolumetric changes to sustain functional recovery.

It must be noted that a considerable number of cases included in these studies reported null neuroimaging findings (Abdelnour et al., 2020; Anand et al., 2020; Fernández-Domínguez et al., 2020; Logmin et al., 2020; Manganelli et al., 2020; Palomar-Ciria et al., 2020; Vollono et al., 2020). This was especially the case for those that investigated COVID-19-related encephalitis (Chaumont et al., 2020; Khoo et al., 2020; Pilotto et al., 2020), a condition that was not always confirmed by abnormal cerebrospinal fluid findings and in some cases diagnosed only on the basis of clinical manifestations. Consistently, one of the largest studies here reviewed found no brain abnormalities in about 80% of the cases examined (Pons-Escoda et al., 2020). This may mean either that the majority of older patients does not experience neurological complications or that functional brain alterations, rather than structural ones, might represent the predominant neural consequences of COVID-19 infection as suggested by hypoperfusion detected by means of PET and functional MRI. However, it is also possible that COVID-19 infection may mainly cause microstructural damage, at least at the acute/early stage, as suggested by the fact that macroscopic alterations were less common than microscopic ones in neuropathological case descriptions. The detection of microstructural damage can be improved by the use of techniques such as diffusion MRI and by the use of 7T MRI scanners that enable greater image resolution. As of December 2020, however, brain imaging in COVID-19 cases has mainly served a clinical purpose and at present there are no studies that have explored microstructural brain features using a research-led approach.

Consistently, neuropathological examinations have also highlighted macroscopic brain injuries of predominantly vascular origin across all brain regions, with a heterogeneous pattern unable to clarify aetiology and tease apart new phenomena from pre-existent comorbidities. Indeed, in almost half of the cases reviewed no macroscopic abnormalities were reported (Bradley et al., 2020; Buja et al., 2020; Matschke et al., 2020; Remmelink et al., 2020; Youd and Moore, 2020). Microscopic examinations, instead, revealed a wide multiplicity of pathological processes found in the majority of cases. In particular, widespread WM damage has been observed as axonal loss, demyelination, and lesions (Jaunmuktane et al., 2020; Kantonen et al., 2020; Reichard et al., 2020) along with WM inflammation (Al-Dalahmah et al., 2020). Consistently, multiple scattered cerebrovascular injuries have been found especially in WM (Bradley et al., 2020; Hernández-Fernández et al., 2020; Reichard et al., 2020; Remmelink et al., 2020). Moreover, neuronal damage and microglial activation have been detected across several brain areas, but especially in the medial temporal lobe (Al-Dalahmah et al., 2020; Reichard et al., 2020), the brainstem, the olfactory bulb and the orbitofrontal cortex (Al-Dalahmah et al., 2020; Bulfamante et al., 2020; Matschke et al., 2020). Such neuropathological findings appear particularly interesting, since they seem to suggest that COVID-19 can induce neural damage particularly in a series of brain structures directly connected or proximal to the olfactory areas, also observed in some cases with neuroimaging assessment (Le Guennec et al., 2020; Lin et al., 2020). This scenario is in line with the hypothesis that infection may spread to the central nervous system through the olfactory epithelium, as for instance demonstrated in a mouse model exposed to Middle-East respiratory syndrome coronavirus (Li et al., 2016). In this respect, the olfactory bulb has already been proposed as the neural point of entry for toxic proteins at the basis of certain neurodegenerative conditions (Rey et al., 2018). Moreover, the structures specialised in the processing of olfactory stimuli are tightly coupled with the mediotemporal lobe. In fact, the piriform cortex projects to the hippocampus *via* the entorhinal and perirhinal cortices (Vismer et al., 2015). These connexions play a central role in the early stages of AD, because TAU pathology is known to spread from cell to cell (Vogels et al., 2020) and the olfactory bulb harbours neurofibrillary pathology already during the transentorhinal Braak stages of AD (Tsuboi et al., 2003). On similar grounds, the mediotemporal lobe would be a prime candidate as target of a COVID-19 axonal propagation originating from the olfactory bulb. Although as a speculation, this mechanism might be at the basis of the mediotemporal involvement described in the MRI and PET case series illustrated above (Delorme et al., 2020; Guedj et al., 2020; Helms et al., 2020a,b; Hosseini et al., 2020; Novi et al., 2020). Additionally, although not all the neuropathological studies detected the presence of COVID-19 in samples of neural tissue, it appears that COVID-19 may be able to penetrate the brain. In fact, viral genetic material has been found mainly in the olfactory bulb (Al-Dalahmah et al., 2020; Bulfamante et al., 2020; Matschke et al., 2020; Menter et al., 2020) and, to a more limited extent, in the frontal lobe (Bulfamante et al., 2020; Matschke et al., 2020) and brainstem (Bulfamante et al., 2020; Matschke et al., 2020; Menter et al., 2020). Since both COVID-19 genetic material (Matschke et al., 2020) and signs of neuronophagia (Lee et al., 2021) were detected in nuclei of the cranial nerves, especially of the vagus nerve, this has been suggested as an alternative route enabling retrograde invasion of the CNS (Bulfamante et al., 2020). In fact, the vagus nerve innervates most abdominal organs, including the lungs. Through the vagal sensory innervation of the alveolar epithelium, the virus could reach the dorsal vagal complex in the brainstem and generate multiple autonomic dysfunctions (Rangon et al., 2020). However, it has also been hypothesised that viral detection in CNS tissue can be due to contamination with blood rich in viral material, especially in cases of cerebrovascular damage (Al-Dalahmah et al., 2020). In support of this hypothesis, the presence of COVID-19 in the CNS was not associated with severity of neuropathology in the study by Matschke et al. (2020), suggesting that the neural alterations observed in patients may be the result of a combination of both direct (i.e., damage to CNS tissue caused by the virus itself) and indirect processes triggered by COVID-19, e.g., neuroimmune stimulation, systemic infection and haematogenous dissemination (Riederer and Ter Meulen, 2020). Therefore, definite conclusions on the spatial distribution and the type of impact, either direct or indirect, of COVID-19 throughout the brain (especially in structures other than those reported by the few neuropathological studies available to date) cannot be yet drawn due to methodological limitations of the available studies.

A large number of publications has described the co-occurrence of COVID-19 and cerebrovascular events, documented by neuroimaging. Slightly less than 2/3 (about 64%) of the patients belonging to this category presented with evidence of ischaemic infarctions. This percentage is not dissimilar from the epidemiological proportion of ischaemic strokes, i.e., equal to ~58% (Shiber et al., 2010), indicating that COVID-19 does not seem to alter this overall proportion. However, COVID-19 infection appears to pose a greater risk of ischaemic stroke to patients than infection by Influenza (1.6 vs. 0.2%) (Merkler et al., 2020). Although these studies appear to consolidate an association between viral infection and stroke, patients presenting with cerebrovascular damage were a small part of the hospitalised patients and an even smaller part of all symptomatic patients. The description of these 84 cases details an extremely heterogeneous picture, with all regions of the brain that appear to be susceptible to adverse acute events. Aetiological variability was also observed, with a number of cases presenting with mixed ischaemic-haemorrhagic or haemorrhagic-encephalopathic profiles. Although, to date, no definite framework has been formulated to account for a definite and established link between COVID-19 and cerebrovascular events, the evidence so far collected indicates that multiple mechanistic avenues are at play. Processes ascribable to a hypercoagulability state, encephalopathy, vasculitis, and cardiomyopathy seem to play a central role (Spence et al., 2020), including increased risk of thromboembolic complications (Lodigiani et al., 2020). It is important to remark that the evidence so far documented has been obtained in a period of acute crisis during which clinical work has taken priority over medical research. Under these circumstances the cause-effect and temporal relationships between viral infection and neurological dysfunction has been challenging to verify or investigate (Radmanesh et al., 2020a). As a consequence, it is not possible to draw a separating line between neuroimaging- and pathology-based consequences of the virus and other relevant variables that are premorbid or contingent.

Interpretations of the neuroimaging and neuropathological findings in older adults with COVID-19 must take into account a series of additional potential limiting factors. First, some of the patients included in the papers reviewed had prior neurological conditions (e.g., epilepsy), while other studies focussed only on individuals who presented with neurological signs and symptoms. Second, often neuroimaging examinations were carried out on the most severe cases only. Third, equivalence between MRI and CT examinations is unclear, since it appears highly likely that these techniques provide complementary information and are more suitable to detect different types of neural injuries. Fourth, location of neural injuries was not always fully documented by all studies, some of which were excluded due to the absence of precise topographical details about neurological damage. This selection approach might have potentially steered the results of this review mainly towards studies reporting topographical information. Fifth, an additional point on regional differences relates to 11 out of 16 neuropathological studies examining the whole brain. Some studies focussed their analyses on olfactory, frontal, and brainstem areas only (Bulfamante et al., 2020; Matschke et al., 2020; Lee et al., 2021), while Hanley et al. (2020) limited their analyses to eight non-specified areas, leading to a potential over-representation of such areas among the currently available results. In fact, the first neuropathological studies may have focused on a limited number of regions to generate knowledge on COVID-19 impact on the central nervous system more quickly (Glatzel, 2020) and may have been mainly led by the dominant hypothesis suggesting that viral spreading into the CNS might be mediated by olfactory neurons (Riederer and Ter Meulen, 2020). Sixth, the causal relationship between COVID-19 infection and some of the neural changes observed has not been addressed, since only some studies distinguished acute and prior neural findings. Finally, no paper focussed exclusively on the effects of COVID-19 on patients with neurological conditions, although some papers included people with conditions such as AD, Parkinson's disease and multiple sclerosis.

In conclusion, the evidence in support of a link between COVID-19 and acute neurological abnormalities is abundant but is characterised by wide heterogeneity. It is still undetermined whether the long-term effects of this infection will be limited to the sequelae of acute neural dysfunction or whether additional mechanisms will play a part in the long term. While it is possible to speculate about the long-term neurofunctional consequences derived from the chronic evolution of acute events, it is still unknown whether other, sub-clinical events may be exacerbated by COVID-19 infection. It is thus possible that any long-term effect may be the result of a complex interplay of chronic alterations and subtle and insidious mechanistic changes that are clinically negligible *per se*, but that may contribute to increase neural vulnerability. It is also unknown whether any effect on the nervous system will be relevant to patients who have not undergone a serious disease phase (i.e., non-hospitalised and asymptomatic patients who may have been only subjected to silent changes, e.g., microscopic ischaemic events). We expect that the link between COVID-19 and neurologically-informed cognitive/psychiatric dysfunction will be better elucidated when concrete data are available and when these are collected and analysed based on the formulation of research-based hypotheses. In the meantime, however, a systematic review of the regions of the brain that are targeted in the acute phase suggests that multiple neural systems (e.g., brain networks) may be exposed to a virus-related vulnerability. These are large-scale functional patterns that sustain high-order mental abilities such as memory and attention/executive functioning, cognitive domains that are also negatively influenced by the ageing process and by major neurodegenerative conditions, suggesting that due their high susceptibility to viral-related additional pathological processes, long-term post-COVID cognitive/neuropsychiatric sequelae might manifest with potentially more severe/more rapidly progressing phenotypes in older adults. Indeed the findings of this review seem to parallel some preliminary findings indicating that older adults with reduced connectivity pre-infection (from scans acquired on average 3 years prior to infection) in regions within the networks supporting attention and executive functioning are at increased risk of COVID-19 infection (Abdallah, 2021), but at the same time indicating a potentially specific pre-existing neural vulnerability of older individuals that significantly lowers their brain resilience potential. Finally, it is also important to point out that, other than reporting preliminary findings, the conclusions of this systematic review are also exclusively based on single-case descriptions and case series. Although the large number of clinical cases linking COVID-19 and brain alterations is, *per se*, sufficient to discard the possibility of this association being anecdotal, the description of this link is still, to some extent, *anecdotal*, and is still conceptually distant from the gold standard of “evidence-based” clinical research. Evidence-based medicine is at the basis of modern healthcare policies, and for this reason, considerable progress is warranted in this field of research, in order to strengthen the nature of the data in support of the above link.

## Data Availability Statement

The original contributions presented in the study are included in the article/supplementary material, further inquiries can be directed to the corresponding author/s.

## Author Contributions

AV conceived this study, reviewed, and finalised the manuscript. RM and MDM designed this study, carried out the literature search, selected the papers for inclusion, summarised the literature findings, and wrote this manuscript. PGI critically reviewed this manuscript. All authors approved the final version of this manuscript.

## Conflict of Interest

The authors declare that the research was conducted in the absence of any commercial or financial relationships that could be construed as a potential conflict of interest.
